# β1 integrins regulate cellular behaviour and cardiomyocyte organization during ventricular wall formation

**DOI:** 10.1093/cvr/cvae111

**Published:** 2024-05-24

**Authors:** Lianjie Miao, Yangyang Lu, Anika Nusrat, Luqi Zhao, Micah Castillo, Yongqi Xiao, Hongyang Guo, Yu Liu, Preethi Gunaratne, Robert J Schwartz, Alan R Burns, Ashok Kumar, C Michael DiPersio, Mingfu Wu

**Affiliations:** Pharmacological and Pharmaceutical Sciences, College of Pharmacy, University of Houston, Houston, TX 77204-5039, USA; Pharmacological and Pharmaceutical Sciences, College of Pharmacy, University of Houston, Houston, TX 77204-5039, USA; Pharmacological and Pharmaceutical Sciences, College of Pharmacy, University of Houston, Houston, TX 77204-5039, USA; Pharmacological and Pharmaceutical Sciences, College of Pharmacy, University of Houston, Houston, TX 77204-5039, USA; Department of Biology and Biochemistry, University of Houston Sequencing and Gene Editing Core, University of Houston, Houston, TX 77204-5001, USA; Pharmacological and Pharmaceutical Sciences, College of Pharmacy, University of Houston, Houston, TX 77204-5039, USA; Pharmacological and Pharmaceutical Sciences, College of Pharmacy, University of Houston, Houston, TX 77204-5039, USA; Pharmacological and Pharmaceutical Sciences, College of Pharmacy, University of Houston, Houston, TX 77204-5039, USA; Department of Biology and Biochemistry, University of Houston Sequencing and Gene Editing Core, University of Houston, Houston, TX 77204-5001, USA; Department of Biology and Biochemistry, University of Houston Sequencing and Gene Editing Core, University of Houston, Houston, TX 77204-5001, USA; College of Optometry, University of Houston, Houston, TX 77204-2020, USA; Pharmacological and Pharmaceutical Sciences, College of Pharmacy, University of Houston, Houston, TX 77204-5039, USA; Department of Surgery, Albany Medical College, Albany, NY 12208, USA; Pharmacological and Pharmaceutical Sciences, College of Pharmacy, University of Houston, Houston, TX 77204-5039, USA

**Keywords:** Trabecular morphogenesis, Ventricular wall specification, Integrin-extracellular matrix interaction, Cellular organization, Cell behaviour

## Abstract

**Aims:**

The mechanisms regulating the cellular behaviour and cardiomyocyte organization during ventricular wall morphogenesis are poorly understood. Cardiomyocytes are surrounded by extracellular matrix (ECM) and interact with ECM via integrins. This study aims to determine whether and how β1 integrins regulate cardiomyocyte behaviour and organization during ventricular wall morphogenesis in the mouse.

**Methods and results:**

We applied mRNA deep sequencing and immunostaining to determine the expression repertoires of α/β integrins and their ligands in the embryonic heart. Integrin β1 subunit (β1) and some of its ECM ligands are asymmetrically distributed and enriched in the luminal side of cardiomyocytes, and fibronectin surrounds cardiomyocytes, creating a network for them. *Itgb1*, which encodes the β1, was deleted via *Nkx2.5^Cre/+^* to generate myocardial-specific *Itgb1* knockout (B1KO) mice. B1KO hearts display an absence of a trabecular zone but a thicker compact zone. The levels of hyaluronic acid and versican, essential for trabecular initiation, were not significantly different between control and B1KO. Instead, fibronectin, a ligand of β1, was absent in the myocardium of B1KO hearts. Furthermore, B1KO cardiomyocytes display a random cellular orientation and fail to undergo perpendicular cell division, be organized properly, and establish the proper tissue architecture to form trabeculae. Mosaic clonal lineage tracing showed that *Itgb1* regulates cardiomyocyte transmural migration and proliferation autonomously.

**Conclusion:**

β1 is asymmetrically localized in the cardiomyocytes, and some of its ECM ligands are enriched along the luminal side of the myocardium, and fibronectin surrounds cardiomyocytes. β1 integrins are required for cardiomyocytes to attach to the ECM network. This engagement provides structural support for cardiomyocytes to maintain shape, undergo perpendicular division, and establish cellular organization. Deletion of *Itgb1* leads to loss of β1 and fibronectin and prevents cardiomyocytes from engaging the ECM network, resulting in failure to establish tissue architecture to form trabeculae.

## Graphical Abstract

**Graphical Abstract:**
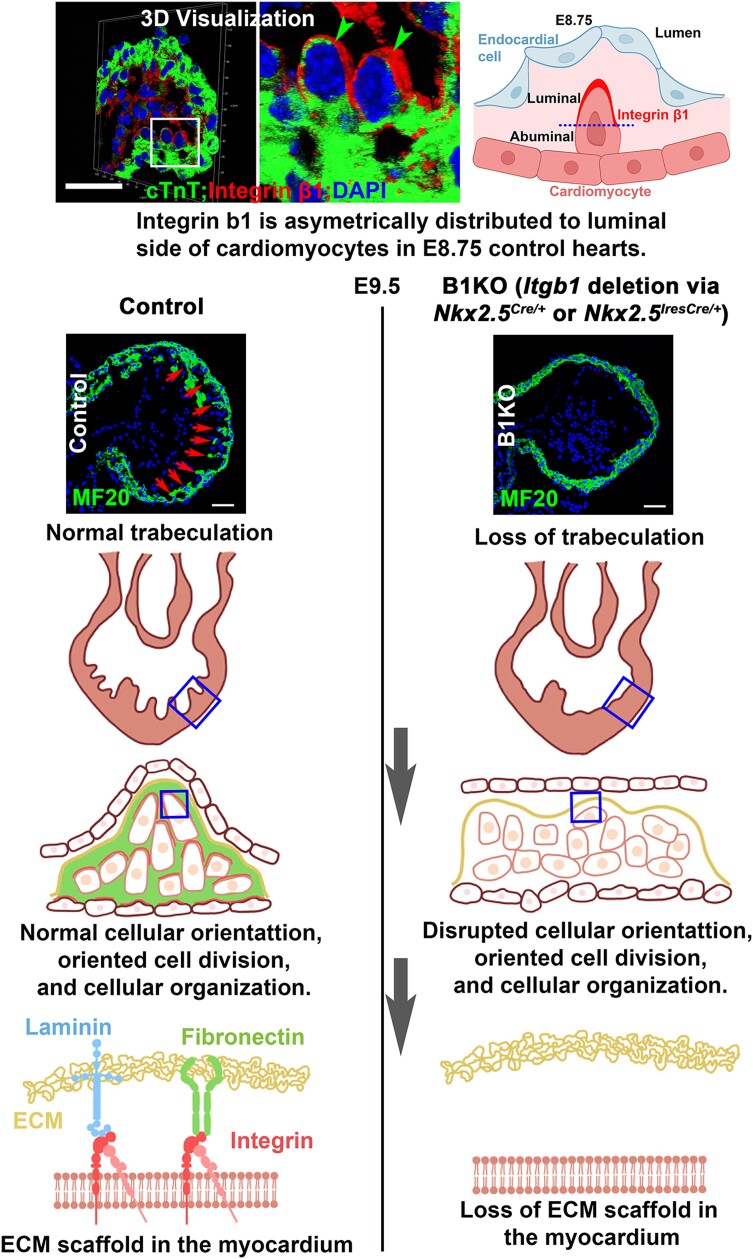

**Time of primary review: 30 days**

## 1. Introduction

The heart is the first functional organ formed in mammalian embryonic development.^1^ During cardiac morphogenesis, cardiac progenitor cells from the cardiac crescent migrate toward the ventral midline to form a linear heart tube with a smooth inner surface.^2^ When the heart tube undergoes looping, the myocardium along the outer curvature of the tube grows inward and forms sheet-like structures that extend from the myocardium,^2^ which are the newly initiated trabeculae.^3^ As the early embryonic heart does not have a coronary circulatory system to perfuse itself, the sheet-like trabeculae function to increase surface area to facilitate nutrients and oxygen exchange. A lack of trabeculation will result in less availability of oxygen and nutrients in the myocardial tissue and end with embryonic lethality, while excess trabeculation will cause left ventricular non-compaction cardiomyopathy in humans.^4^ The trabecular formation is a multistep process that includes but is not limited to, trabecular initiation, specification, growth, and compaction.^5,6^ Recent works show that cardiomyocytes in the monolayer myocardium display polarity, and proper cellular behaviour such as oriented cell division (OCD) and directional migration of the cardiomyocytes in the monolayer myocardium contribute to trabecular initiation in an N-Cadherin dependent manner.^6–11^ However, the underlying mechanisms regulating cell behaviour and organization during trabecular morphogenesis and ventricular wall formation require further study. The cells in the monolayer myocardium are not typical cardiomyocytes, as gap junctions and intercalated discs are not fully formed yet. They present some features of epithelial cells as they display polarity and squamous or cuboidal shape, but they are not epithelial cells, as these cells do not have the typical tight junctions and desmosomes.^12^ So, how these cells become organized to establish the tissue architecture of trabeculae and ventricular wall is unknown.

Integrins are transmembrane heterodimeric proteins consisting of an α and a β subunit, each with a large extracellular domain, a single-pass transmembrane domain, and a cytoplasmic domain.^13^ Integrins are the major cell surface receptors for adhesion to the extracellular matrix (ECM) and mediate both inside-out and outside-in signal transduction pathways that control various cell functions, including proliferation, survival, migration, and gene expression.^13,14^ We found that the β1 integrin subunit (β1), encoded by *Itgb1,* is highly expressed in all cardiac cell types. Global deletion of *Itgb1* is lethal at the pre-implantation stage.^15,16^ Endothelial-specific knockout of *Itgb1* disrupts cell polarity and arteriolar lumen formation^17^ and results in severe vascular defects and lethality at E10.5.^18,19^ Cardiomyocyte-specific knockout of the *Itgb1* via *Mlc-2vCre* results in myocardial fibrosis and cardiac failure in the adult heart,^20^*Itgb1* deletion via the *cTnT-Cre* or *Xmlc2^Cre/+^* causes myocardial rupture at about E14.5,^21,22^ and *Itgb1* deletion via the transgenic *Nkx2.5Cre* line perturbs cardiomyocyte proliferation with progressive cardiac abnormalities seen toward birth.^23^ Since the four Cre lines ablate *Itgb1* at relatively later stages, the requirement for β1 integrins at earlier stages of trabecular and ventricular morphogenesis is unclear. This study found that *Itgb1* deletion at an early stage via *Nkx2.5^Cre/+^*^24^ or *Nkx2.5^IRES-Cre/+^*^25^ causes distinct defects.

This study aimed to determine whether and how β1 integrins regulate early cardiomyocyte behaviour and organization during ventricular wall morphogenesis in the mouse. We conclude that β1 integrins mediated-cardiomyocyte–ECM interactions provide a structural framework and a microenvironmental niche that are critical to proper cellular orientation, behaviour, organization, and cell specification. Targeted cardiomyocyte loss of β1 integrins results in trabeculation and ventricular wall specification defects. This study provides a basis for understanding the pathogenesis of trabeculation defects and how ECM regulates ventricular wall morphogenesis.

## 2. Methods

### 2.1 Mouse lines

Mouse lines of *Itgb1^fl/fl^*,^26^*Notch1^fl/fl^*,^27^*Rosa26Cre^ERT2^* (*iCre*),^28^*Rosa26-Confetti* (*Conf*),^29^*Rosa26-mTmG* (*mTmG*),^30^*Nkx2.5^IRES-Cre/+^*,^25^ and *Tie2-Cre*^31^ were purchased from Jackson Lab. Dr. Robert Schwartz provided *Nkx2.5^Cre/+^*^24^ mice. All animal experiments were approved by the Institutional Animal Care and Use Committee (IACUC) at the University of Houston and performed according to the NIH Guide for the Care and Use of Laboratory Animals.

Other materials and methods are briefly described in the results, and their details are provided in the Supplementary material online, *File*.

## 3. Results

### 3.1 β1 integrins and their ligands are asymmetrically localized in the myocardium

**Figure 1:**
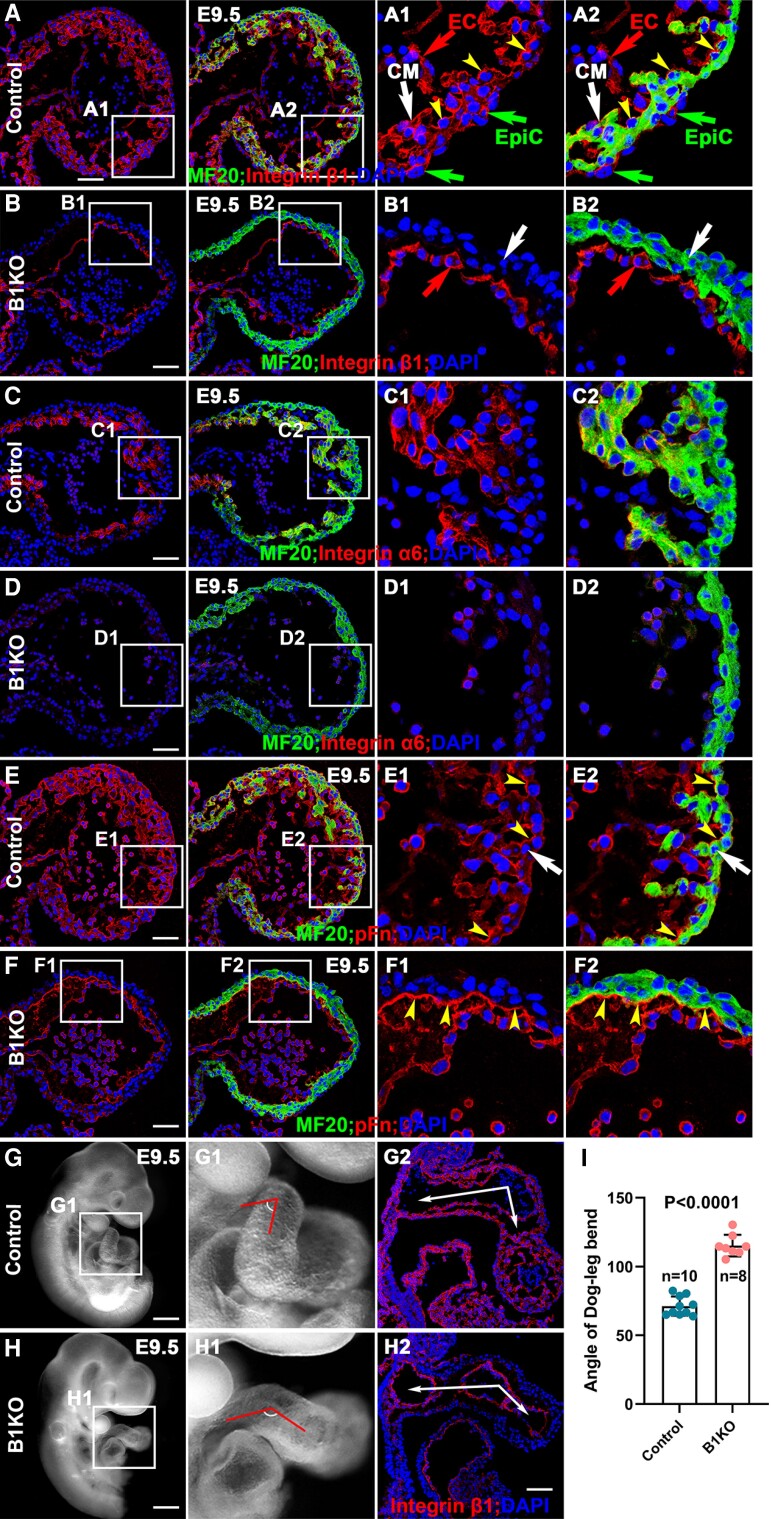
Expression of β1 and its ligands in the E9.5 hearts. (*A*, *A1, A2*) In E9.5 hearts, β1 is abundantly expressed in cardiomyocytes (CM, arrows) and endocardial cells (EC, arrows) and enriched in the luminal side of the cardiomyocytes. The MF20 negative cells that attach to the surface of the myocardium are epicardial cells (EpiC, arrows). (*B*, *B1,* and *B2*) *Itgb1* was efficiently deleted from the myocardium but not endocardium by *Nkx2.5^cre/+^*. Integrin α6 subunit is enriched in trabecular cardiomyocytes (*C*, *C1*, and *C2*) but reduced or absent in B1KO hearts (*D*, *D1*, and *D2*). Plasma Fn (pFn) is abundantly expressed in the basal membrane and luminal side (yellow arrows) of the myocardium (white arrows) (*E*, *E1*, and *E2*) and is reduced in the myocardium of B1KO hearts, but remains expressed in the luminal side of the myocardium (*F*, *F1*, and *F2*). (*n* ≥ 3 in *A*–*F*). (*G–I*) The B1KO hearts have a larger rotation angle at the Dog-Leg bend (*n* = 8–10) based on the two-tailed Student’s *t*-test (*I*). Scale bar: 50 μm in (*A–F*), 300 μm in (*G* and *H*).

Integrins are essential to mediate cell–ECM interactions.^32^ A detailed expression repertoire of integrins and their ligands during cardiac morphogenesis is not available.^32^ Therefore, we examined the expression of genes that encode α or β subunits of integrins and their ligands in hearts at E9.5 by mRNA deep sequencing and their expression patterns by immunostaining and RNAScope (see Supplementary material online, *Table S1* and *Figure 1A–F*). Of all the genes that encode the β subunits, *Itgb1* was the most abundantly expressed (see Supplementary material online, *Table S1*) and was expressed in cardiomyocytes, endocardial cells, and epicardial cells based on immunostaining (*Figure 1A* and Supplementary material online, *Figure S1A* and *B*). Surprisingly, β1 was not evenly distributed on the membrane of cardiomyocytes but enriched along the luminal side (see Supplementary material online, *Figure S1A1* and *2*, yellow arrows). This asymmetry was evident when the myocardial layer is about two-cell thick from the images of sections or reconstructed 3D stacks, with the ratio of luminal to abluminal intensity being around 2 (see Supplementary material online, *Figure S1C–G*). The active β1, endocytosed in the cytoplasm, did not show an obvious asymmetric distribution examined at E9.5 (see Supplementary material online, *Figure S1H* and *I*). Of the genes encoding the α subunits, *Itga6* and *Itga5* were the most abundantly expressed (see Supplementary material online, *Table S1*). The integrin α5 subunit (abbreviated as α5), encoded by *Itga5*, was expressed in trabecular and compact cardiomyocytes (see Supplementary material online, *Figure S2A*). In contrast, the integrin α6 subunit (abbreviated as α6), encoded by *Itga6,* was enriched in trabecular cardiomyocytes at both transcriptional and translational levels (*Figure 1C, C1,* and *C2* and Supplementary material online, *Figure S2C*). Integrin ligands, including fibronectin (Fn), collagen, vitronectin, laminin, and vascular cell adhesion molecules, were examined. In the embryonic heart at E9.5, Fn was abundantly expressed, *Col4a1* was the most abundant gene encoding for collagen, and *lama4*, *b1,* and *c1* were the most abundantly expressed genes encoding laminin α, β, and γ isoforms, respectively (see Supplementary material online, *Table S1*). Fn, the ligand for α5β1, occurs in two main forms: plasma Fn, detected by immunostaining with an anti-40 K, and cellular Fn, identified by the alternatively spliced domain A specific antibody.^33^ We found that the plasma Fn was abundantly expressed proximal to the basal cell layer and luminal side of the myocardium. Furthermore, plasma Fn surrounded the individual cardiomyocytes, creating an ECM scaffold for cardiomyocytes (*Figure 1E*). In contrast, the cellular isoform was expressed in the AV canal but not in the ventricles (see Supplementary material online, *Figure S2E*). Laminin α4, β1, and γ1 (Laminin 411), a ligand for α6β1 integrin, was expressed in the basement membrane and asymmetrically enriched on the luminal side of the myocardium (see Supplementary material online, *Figure S2G*), as was collagen IV (see Supplementary material online, *Figure S2I*), which is highly expressed in the heart.^34^

### 3.2 β1 integrins are required for the expression of Fn and α6

To study the functions of β1 integrins in cardiac morphogenesis, the floxed *Itgb1*, encoding β1, was deleted via the *Nkx2.5^Cre/+^* to generate *Nkx2.5^Cre/+^*; *Itgb1^fl/fl^* (B1KO).^26^ The deletion of *Itgb1* in the myocardium was confirmed by the absence of β1 in the myocardium but not in endocardial cells (*Figure 1B*). β1 heterodimerizes with α5 or α6 to bind to Fn and laminin 411, respectively.^13^ As mentioned, genes encoding β1, α5, or α6 integrin subunit, or their ligands, were abundantly expressed in the heart at E9.5 (see Supplementary material online, *Table S1*). We therefore examined if *Itgb1* deletion affected the expression of α5 or α6 subunits and/or their ligands via mRNA deep sequencing and immunostaining. The transcript levels of genes that encode α and β integrins and their ligands were not significantly different between control and B1KO hearts at E9.5 for any genes whose expression BaseMean is larger than 50 based on mRNA deep sequencing (see Supplementary material online, *Table S1*). Immunofluorescence staining revealed α6 and Fn localization to the myocardium (*Figure 1C–F*), and their expression were reduced or absent in B1KO hearts. Fn staining along the luminal side of the myocardium was not affected, suggesting endocardial cells deposited Fn to the cardiac jelly (*Figure 1E*). The Fn surrounding individual cardiomyocytes creates an ECM scaffold in the myocardium (*Figure 1E, 1E1,* and *1E2*), which was absent in B1KO (*Figure 1F, 1F1,* and *1F2*). These data indicate that β1 is required for the stabilization of both α6 and Fn in the myocardium. Other integrin subunits, such as α5, and other ECM ligands, such as collagen IV and laminin, did not display an obviously different pattern in B1KO compared with the controls (see Supplementary material online, *Figure S2A*, *B*, *G,* and *J*). The B1KO died before E11.5 (see Supplementary material online, *Table S2*), a more severe phenotype than the published transgenic Cre line *Nkx2.5Cre* mediated *Itgb1* knockouts,^23^ in which *Itgb1^fl/fl^* was deleted at a later developmental stage, the *Mlc-2vCre* mediated knockout, which results in myocardial fibrosis and cardiac failure in the adult heart,^20^ and the *cTnT-Cre* or *Xmlc2^Cre/+^* mediated knockouts, which display defects in myocardial integrity and died around E14.5.^21,22^ The B1KO hearts display an abnormal OFT morphogenesis with a significantly large angle of Dog-Leg bend (*Figure 1G–I*). In summary, considering that Fn is required for heart morphogenesis,^35^ the absence of α6 and Fn in B1KO hearts suggests that β1 integrins mediated multiple and distinct integrin–ECM interactions, which are collectively essential for heart morphogenesis.

### 3.3 B1KO hearts display defects in trabecular formation and trabecular/compact zone specification

**Figure 2:**
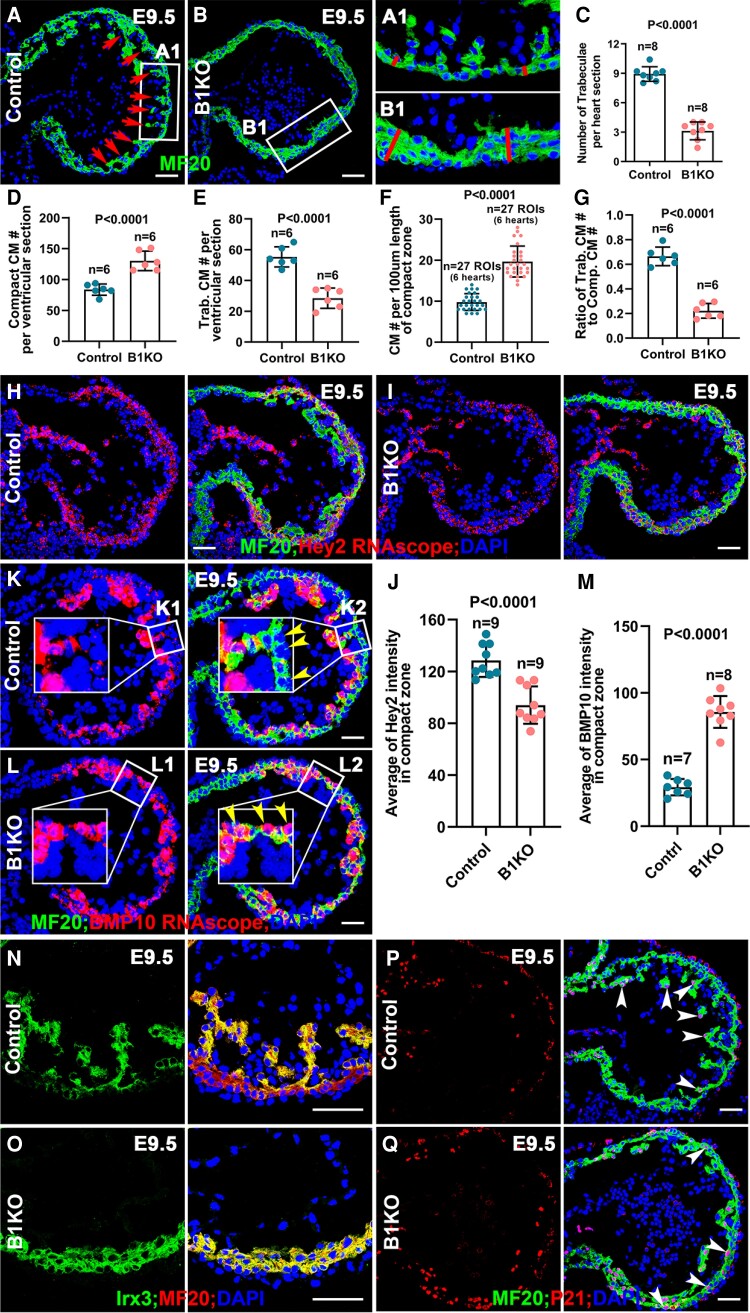
B1KO hearts display defects in trabecular formation and trabecular/compact zone specification. (*A–C*) The E9.5 B1KO hearts display a trabeculation defect, with a significantly smaller number of trabeculae per section and a thicker ventricular wall (*n* = 8). (*D* and *E*) The number of cardiomyocytes in the compact zone is significantly greater, while the number in the trabecular zone is smaller in B1KO compared to the control at E9.5 (*n* = 6). (*F*) The number of cardiomyocytes in region of interest (ROI), 100 μm length of the compact zone, in B1KO is greater than the control (*n* = 27, 27 ROIs from 6 control and B1KO hearts, respectively). (*G*) The ratio of trabecular cardiomyocyte number to compact cardiomyocyte number in B1KO is smaller than the control (*n* = 6). (*H* and *I*) *Hey2* is highly enriched in the compact zone of both control and B1KO hearts at E9.5. (*J*) The fluorescence intensity of *Hey2* in compact myocardium is quantified and compared (*n* = 9, nine sections from three control and B1KO hearts, respectively). (*K* and *L*) In the E9.5 control hearts, *Bmp10* is highly enriched in the trabecular cardiomyocytes but not compact cardiomyocytes. In the E9.5 B1KO hearts, *Bmp10* is also highly enriched in some compact cardiomyocytes. (*M*) The fluorescence intensity of *Bmp10* in compact myocardium is quantified and compared (*n* = 7–8, 7, and eight sections from three control and B1KO hearts, respectively). (*N* and *O*) At E9.5, Irx3 is enriched in the trabecular zone in control hearts but is expressed in the compact zone in B1KO hearts. (*P* and *Q*) P21 is mainly expressed in the trabecular cardiomyocytes in control but is also highly expressed in the compact cardiomyocytes in the B1KO hearts at E9.5 (*n* ≥ 3 in each experiment of (*A–Q*)). Scale bar: 50 μm. The statistic comparison method in (*C*–*G*) and (*J* and *M*) is two-tailed Student’s *t*-test.

**Figure 3:**
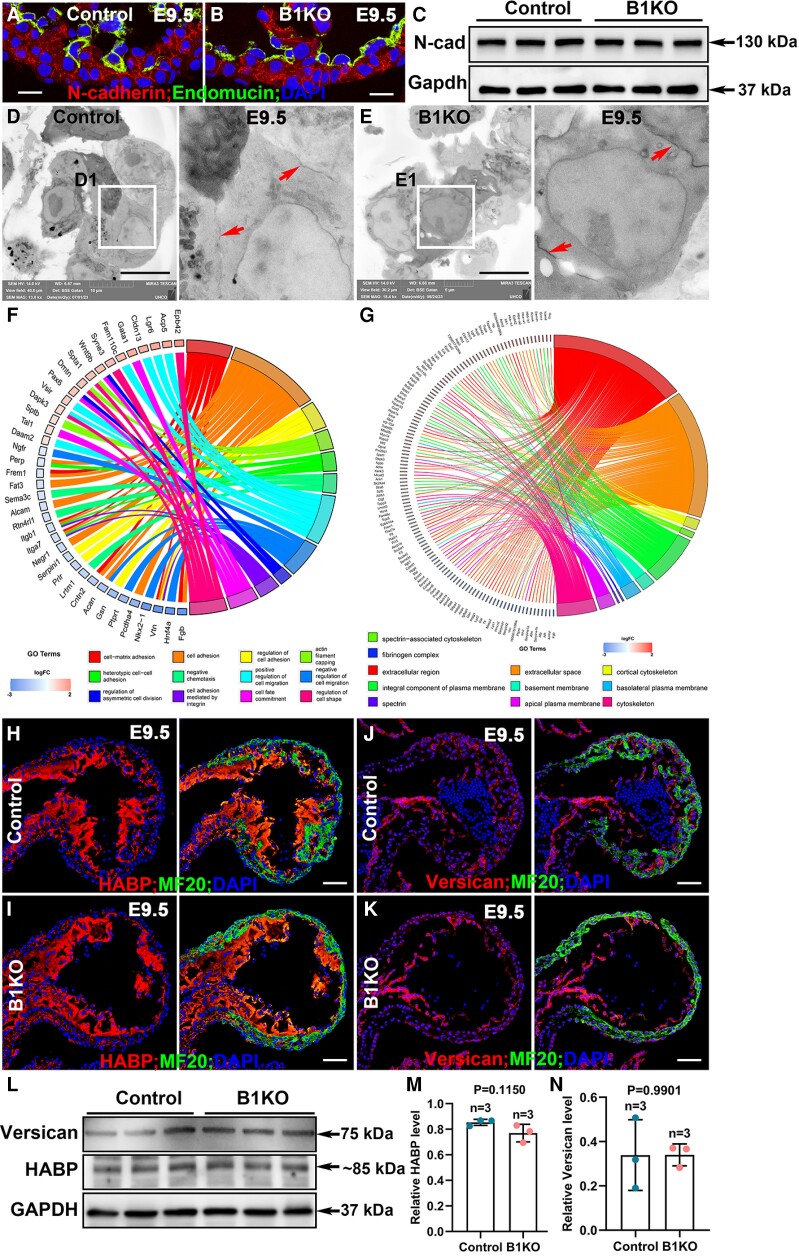
Cell–ECM interactions but not adherens junction-mediated cell–cell interactions were affected. (*A–C*) Adherens junction was not changed in the E9.5 B1KO hearts compared with control hearts, indicated by N-cadherin expression pattern and protein level (*n* = 3). (*D* and *E*) Based on electron microscopy, cell–cell junctions indicated by red arrows in D1 and E1 did not show an obvious difference between control and B1KO hearts (*n* = 3) at E9.5. (*F* and *G*) GO analysis from mRNA deep sequencing using E9.5 hearts showed the affected cellular components and biological processes in B1KO hearts (*n* = 3). (*H–N*) The major cardiac jelly components, HABP and versican, did not show a significant difference between control and B1KO hearts at E9.5 based on immunostaining and western blot (*n* = 3) via the two-tailed Student’s *t*-test. Scale bar: 10 μm in (*A* and *B*), (*D* and *E*), 50 μm in (*H*–*K*).

Trabeculae are sheet-like structures extending from the myocardium to the heart lumen and function to increase surface area when the coronary system is not yet established.^36^ The B1KO hearts display a significantly smaller number of trabeculae per section examined at E9.5, suggesting a trabeculation defect (*Figure 2A–C*); however, the compact zone of B1KO is 2.4 times thicker than the control (*Figure 2A1* and *B1*). We also examined the trabeculation defects in the B1KO mediated by *Nkx2.5^IRES-Cre/+^*, in which the IRES-Cre is inserted to the 3′UTR of *Nkx2.5*.^25^ Consistently, these B1KO hearts display a trabeculation defect (see Supplementary material online, *Figure 3A–C*). The heterozygous *Nkx2.5^IRES-Cre/+^* hearts and homozygous *Nkx2.5^IRES-Cre/IRES-Cre^* hearts did not show obvious trabeculation defects, suggesting that the trabeculation defects in B1KO are not due to the Nkx2.5 expression level (see Supplementary material online, *Figure S3D–G*). We then quantify the number of cardiomyocytes in B1KO and control hearts. The number of cells in the compact zone in B1KO is significantly greater than the control, while the number of cells in the trabecular zone in B1KO is lower; the ratio of the number of trabecular cardiomyocytes to that of compact cardiomyocytes is significantly lower in B1KO (*Figure 2D–G*) (*n* = 6 hearts at E9.5). The thicker compact zone with more cells and the reduced number of trabeculae with fewer cells in B1KO suggest that cardiomyocytes failed to be properly organized and distributed between trabecular and compact zones in B1KO. The trabeculation defects were further confirmed in B1KO at E10.5 (see Supplementary material online, *Figure S3H–J*). Trabecular cardiomyocytes are more differentiated than compact cardiomyocytes,^3^ with trabecular cardiomyocytes exhibiting a lower proliferation rate and being more molecularly mature than cardiomyocytes of the compact zone.^37^ For example, *p21*, *Nppa, Irx3, Bmp10, Sphingosine 1-phosphate receptor-1*, and *Cx40* are highly expressed in the trabecular zone, while *Tbx20*, *Hey2,* and *N-Myc* are highly expressed in the compact zone.^3,5,7,10,38–40^ The regulation of their specific expression patterns in trabecular and compact zones has not been elucidated. We wished to determine if β1 regulates trabecular/compact zone specification by examining the expression patterns of trabecular and compact zone markers in B1KO hearts. *Hey2* is expressed in the compact zone in both control and B1KO (*Figure 2H* and *J*), but the abundance of *Hey2* mRNA in the compact zone in B1KO was reduced compared to control littermates (*Figure 2H–J* and Supplementary material online, *Figure S4A*). *Bmp10*, a marker for trabecular cardiomyocytes, is expressed in the trabecular cells in the control (*Figure 2K, K1,* and *K2*) but is highly expressed in compact cells in B1KO (*Figure 2L, L1, L2,* and *M*). B1KO hearts display increased staining for *Bmp10* compared to the control (*Figure 2K–M*), and mRNA expression is increased (see Supplementary material online, *Figure S4B*). The increased expression of *Bmp10* in B1KO is supported by the increased staining for Smad1/5/8 phosphorylation in cardiomyocytes (see Supplementary material online, *Figure S4C* and *D*), a readout for *Bmp10*.^41^*Irx3* is expressed in the trabecular zone in the control as anticipated, but it is also expressed in the compact zone in B1KO (*Figure 2N* and *O*). Consistently, *Nppa* is enriched in trabecular zone in the control but is highly expressed in both the trabecular and compact zones in B1KO (see Supplementary material online, *Figure S4E* and *F*). P21 was mainly expressed in the trabecular cardiomyocytes in control but was expressed in both trabecular and compact cardiomyocytes with a significantly greater percentage in B1KO compared to the control (*Figure 2P* and *Q* and Supplementary material online, *Figure S4G*). The abnormal expression patterns of trabecular and compact zone-specific markers in B1KO hearts suggest a trabecular and compact zone specification defect and implicate β1 integrins in regulating ventricular wall specification.

Since the trabecular cardiomyocytes are more molecularly mature than cardiomyocytes of the compact zone,^37^ the specification defects in B1KO suggested that the B1KO hearts may show an early maturation defect. We examined sarcomere organization in control and B1KO hearts at E9.5. The sarcomeric array formation in the trabecular zone is more organized than that of the compact zone in a control heart (see Supplementary material online, *Figure S4H*). The sarcomeric array formation in the compact zone in B1KO is more organized than that of the control but less organized than cells in the trabecular zone of the control (see Supplementary material online, *Figure S4H* and *I*). This was further confirmed by electron microscopy (EM), as only the trabecular zone displayed a regular sarcomeric array in control, but the compact zone showed a sarcomeric array in B1KO (see Supplementary material online, *Figure S4J* and *K*), suggesting an early maturation defect.

### 3.4 Genes involved in cell–ECM interactions, but not genes involved in adherens junction-mediated cell–cell interactions, are altered in B1KO hearts

Previous studies show that N-Cadherin, the major component of adherens junctions in the embryonic heart, regulates cellular behaviour and is required for trabecular initiation and ventricular wall morphogenesis.^42,43^ We examined the adherens junctions among cardiomyocytes and found that N-Cadherin localization was not affected by *Itgb1* deletion based on immunostaining (*Figure 3A* and *B*), and the total N-Cadherin protein level was not significantly different between the control and B1KO samples (*Figure 3C* and Supplementary material online, *Figure S5*). Ultrastructural observations suggest cell–cell contacts were not obviously affected (*Figure 3D* and *E*).

We examined the transcriptional levels of genes involved in cell–ECM interaction, cell–cell interaction, and cardiac jelly via mRNA deep sequencing. Three E9.5 control or B1KO hearts were combined as one sample, and three samples of control and B1KO were subjected to mRNA deep sequencing. Compared with control hearts, B1KO hearts showed significant differences in genes that encode cellular components of an extracellular region, extracellular space, basement membrane, fibrinogen complex, spectrin, and other cellular components (*Figure 3F*). Further biological process analyses showed cell-matrix adhesion, heterotypic cell–cell adhesion, regulation of asymmetric cell division, cell migration, cell shape, etc., were significantly different between control and B1KO hearts (*Figure 3G*). The expression of several matrix genes (*Has1*, *Has2*, *Has3*, and *Vcan*) was not altered in B1KO hearts based on mRNA deep sequencing. Major components in cardiac jelly, including versican and hyaluronic acid binding protein (HABP), were also not significantly different when examined by immunostaining and western blot (*Figure 3H–N* and Supplementary material online, *Figure S5*). Together, these findings indicate the trabeculation defect in B1KO was not due to changes in genes that contribute to the cardiac jelly or cell–cell adherens junctions but was more likely due to cell–ECM-mediated interaction.

### 3.5 Cardiomyocytes in B1KO hearts display abnormal cellular organization.

**Figure 4:**
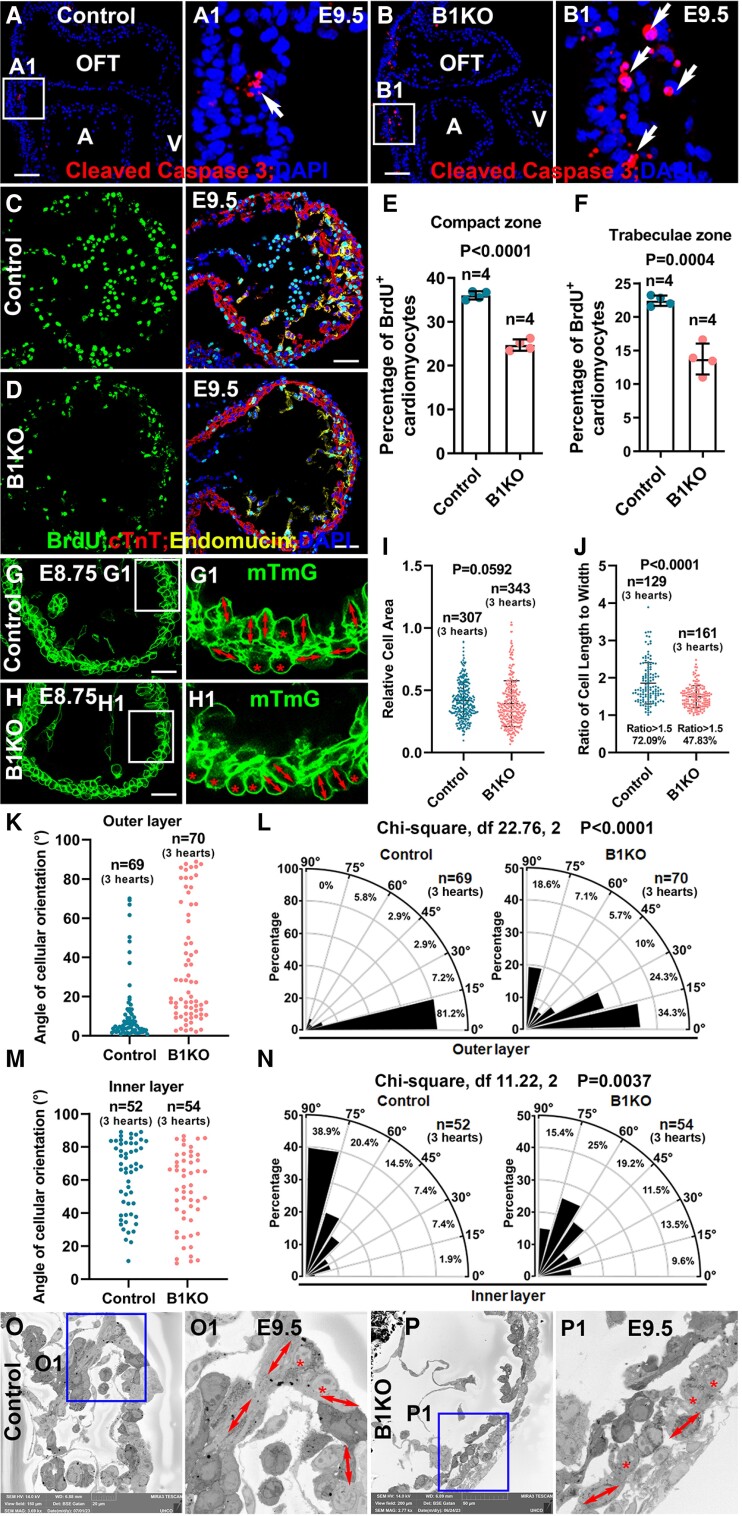
Cardiomyocytes in B1KO display abnormal cellular organization. (*A* and *B*) Immunostaining in the E9.5 B1KO hearts for cleaved caspase 3 reveals that B1KO hearts have more cell death in the OFT but not in the ventricular region compared with control hearts (*n* = 3). (*C–F*) Based on BrdU pulse labelling, the cardiomyocytes in E9.5 B1KO hearts displayed lower proliferation rates than the control (*n* = 4) based on the two-tailed Student’s *t*-test. (*G* and *H*) The mTmG reporter outlined cardiomyocytes in control and B1KO hearts at E8.75, and their cell size and shape were measured (cells for quantification were from three control and three B1KO hearts, respectively). (*I*) Cardiomyocyte size between control and B1KO hearts was not an obvious difference (*n* = 307 cells from three control hearts, *n* = 343 cells from three B1KO hearts) based on the Mann–Whitney test. (*J*) Lower percentage of cardiomyocytes display an orientation in B1KO hearts compared to the control via Mann–Whitney test (quantified cells are from three control and three B1KO hearts, respectively). (*K–N*) Oriented angles of cardiomyocytes in both control and B1KO hearts were measured (*K* and *M*). In the control hearts, most of the cardiomyocytes in the outer layer of the compact zone are oriented parallel to the heart wall, and cardiomyocytes in the inner layer of the compact zone are oriented perpendicularly to the heart wall. In the B1KO hearts, most cells display parallel orientation to the heart wall (*L* and *M*) (quantified cells are from three control and three B1KO hearts, respectively), and the percentages of cells with 0–30°, 30–60°, and 60–90° angle are compared via *χ*^2^ test. (*O–P*) The abnormal cellular orientation in B1KO was confirmed by the EM at E9.5 (*n* =−3). Scale bar: 50 μm in (*A–D*), 20 μm in (*G* and *H*).

β1 is required for cardiomyocyte survival in a mosaic model.^44^ To determine whether cell death causes the trabeculation defects in B1KO, control and B1KO heart sections were stained for an apoptotic marker, cleaved Caspase 3. We found that the B1KO hearts displayed more cleaved caspase 3 positive cells in the OFT region but not in the ventricular region of either control or B1KO hearts (*Figure 4A* and *B*), suggesting cell death was not the cause of trabeculation defects. Next, the proliferation rate was evaluated via BrdU pulse-labelling for 1 h. The cardiomyocytes in both trabecular and compact zones in B1KO hearts displayed a significantly reduced proliferation rate compared to the control at E9.5 and E10.5 (*Figure 4C–F*, Supplementary material online, *Figure S6 A*–*D*). However, the proliferation rates are not significantly different at E8.5 (see Supplementary material online, *Figure S6E–G*), a stage when trabeculae are initiated, suggesting that the trabecular initiation defects in B1KO are not due to cell proliferation. Instead, the reduction of the trabeculae, coupled with a thicker compact zone containing more cardiomyocytes in the compact zone in B1KO hearts (*Figure 2A–G*), suggested that the impaired trabecular formation likely stems from abnormal cellular behaviour and cellular organization.

The cells in the myocardium at early embryonic stage are not mature cardiomyocytes. How these cells become organized to form trabeculae is unknown. We hypothesize that the ECM scaffold provides a structural framework for cardiomyocytes to be properly organized and to support cell shape, OCD, and migration. We, therefore, measured and compared the size of cardiomyocytes in control and B1KO hearts. Specifically, the cell areas of individual cells in multiple sections were measured based on the membrane identified by membrane-localized GFP or RFP. We found that the cell size was not significantly different between the control and B1KO at about E8.75 when the myocardium contained two layers of cells (*Figure 4G–I*). We next evaluated the cell shape and cellular orientation of cardiomyocytes in the inner and outer layers of the myocardium. The ratio of length to width of cardiomyocytes was measured and quantified in control and B1KO hearts. A cell with a ratio larger than 1.4 was defined as an oriented cell, while those less than 1.4 were quantified as non-oriented cells.^45^ Cells in the embryonic stage are more dynamic, therefore, a more stringent criteria with a ratio of 1.5 was set as the threshold to define an oriented cell. The cellular orientation was measured by the angle between the heart surface and the axis of the oriented cells, as described previously.^7^ We found that more than 72% of the control cells had a ratio larger than 1.5, while less than 48% of the cells in B1KO displayed a ratio larger than 1.5 (*Figure 4J*).

A previous study showed that most myocardial cells in the outer layer display a parallel pattern, while cells in the inner layer display a perpendicular pattern.^42^ Consistently, most control cells (81.2%) in the outer layer display a parallel orientation with an angle of less than 15° (*Figure 4K* and *L*). In contrast, B1KO cells display a random orientation with a significantly larger angle (*Figure 4K* and *L*). 59.3% of control cells in the inner layer were oriented with an angle larger than 60°; 9.3% of the cells were oriented with an angle less than 30° in the control cells (*Figure 4M* and *N*). In contrast, 40.4% of the B1KO cells were oriented with an angle larger than 60°, and 23.1% were oriented with an angle less than 30° (*Figure 4M* and *N*), suggesting a random orientation pattern in B1KO. The abnormal cellular orientation in B1KO was further confirmed by EM (*Figure 4O, O1, P,* and *P1*). The random orientation of the cells is consistent with disrupted cellular organization and a loss of growth direction toward the heart lumen.

### 3.6 β1 integrins are required for cardiomyocyte OCD and asymmetric cell division

**Figure 5:**
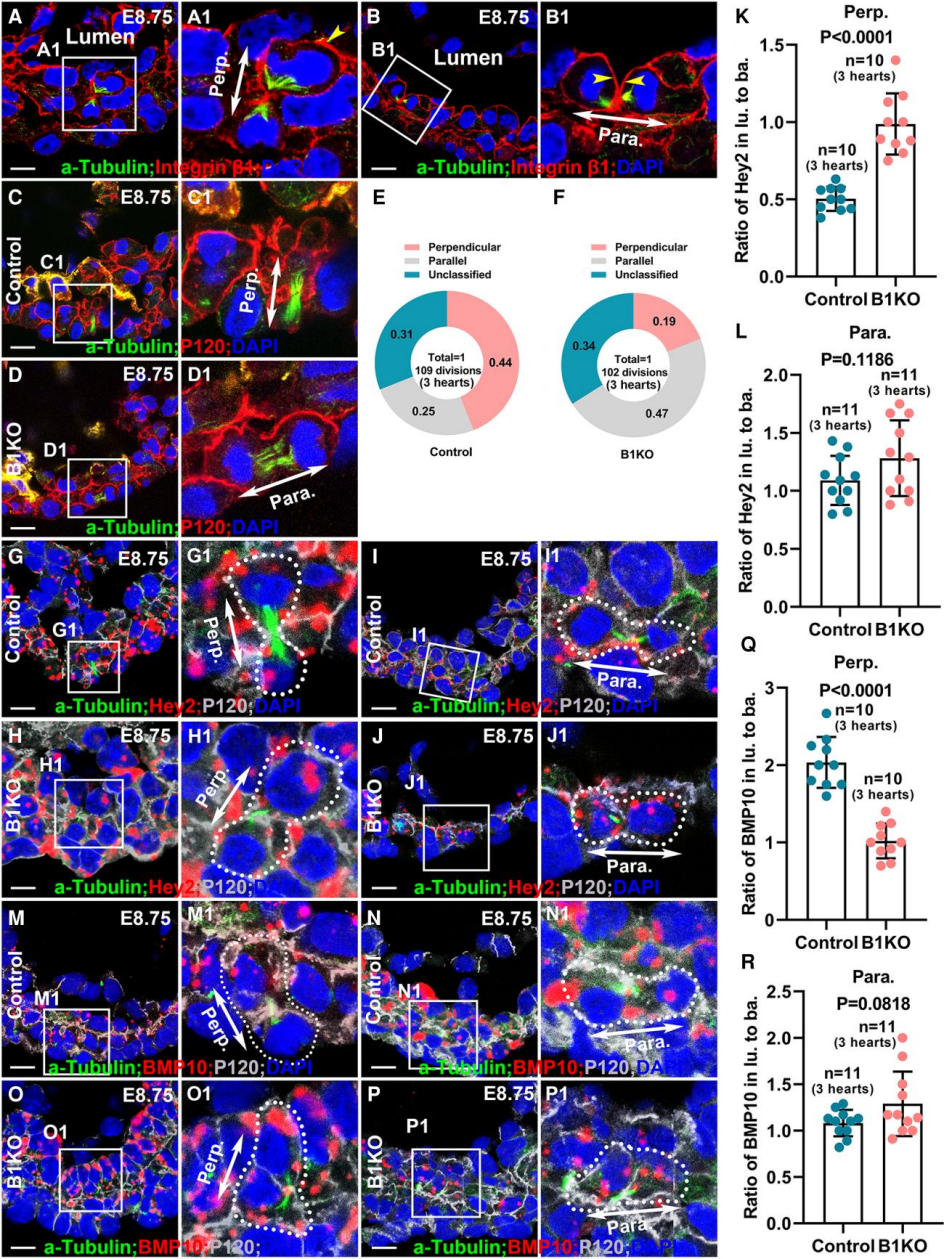
β1 integrins are required for cardiomyocyte OCD and asymmetric cell division. (*A* and *B*) β1 is asymmetrically distributed in dividing cardiomyocytes in E8.75 hearts, with the luminal side of the daughter cells expressing more β1 in both perpendicular and parallel divisions. (*C–F*) Cardiomyocytes in B1KO and control heart undergo different division patterns with a higher percentage of perpendicular divisions in control hearts but a higher percentage of parallel divisions in B1KO hearts at E8.75. (*G–R*) In the dividing cardiomyocytes of control hearts, *Hey2* or *BMP10* was asymmetrically distributed to two daughter cells during perpendicular divisions at telophase or two-cell stage (*G*, *M*, *K*, *Q*) and was symmetrically distributed to two daughter cells during parallel divisions at telophase or at two-cell stage (*I*, *N*, *L*, *R*), but in the B1KO hearts, *Hey2* or *BMP10* was symmetrically distributed to two daughter cells during both perpendicular (*H*, *O*, *K*, *Q*) and parallel (*J*, *P*, *L*, *R*) divisions in E8.75 hearts. Cells for quantification in (*G–R*) were from three control and three B1KO hearts, respectively. The statistic comparsion method in *K*, *L*, *Q*, and *R* is two-tailed Student’s *t*-test. Scale bar: 10 μm.

Previous studies demonstrate that OCD and directional migration contribute to trabecular initiation and trabecular specification in mice, which differs from the mechanism of apical constriction-mediated directional migration contributing to trabecular initiation in zebrafish.^6,7,12,46,47^ We asked whether the β1 integrins are required for trabecular initiation. We first examined if β1 was asymmetrically distributed in dividing cardiomyocytes by staining for β1 and acetylated α-Tubulin to identify the mitotic spindles. We found that β1 is enriched in the membrane along the luminal side of the perpendicular dividing cells at telophase (*Figure 5A*) and is localized to the luminal side of both daughter cells in parallel dividing cells (*Figure 5B*). This asymmetric distribution of β1 suggests that β1 integrins might be required for OCD and asymmetric cell division. To determine if the cardiomyocytes in B1KO display defective OCD, ∼E8.75 control and B1KO hearts were stained for acetylated α-Tubulin and P120 (an adherens junction-associated protein that marks the cardiomyocyte membrane). We measured the spindle orientations of mitotic cardiomyocytes of the left ventricular myocardium, and only the cells at anaphase or early telophase with both centrosomes in the same focal plane were quantified.^7,48^ We found most mitotic cells displayed either perpendicular orientation (44%, *n* = 109) with an angle greater than 60° relative to the heart surface (*Figure 5C*) or parallel orientation (25%, *n* = 109) with a spindle angle less than 30° (*Figure 5D*). However, cardiomyocytes in B1KO undergo a significantly different division pattern and most of the divisions were parallel with 47% divisions being parallel and 19% being perpendicular (*P* < 0.01, *n* = 102) (*Figure 5E* and *F*). The parallel division pattern may partially explain the thicker compact zone and the absence of trabecula.

Perpendicular-OCD is an extrinsic asymmetric cell division contributing to trabecular specification and this mechanism results in trabecular cardiomyocytes being distinct from cardiomyocytes in the compact zone.^7^ The cues that regulate asymmetric cell division are unknown. The asymmetric distribution of β1 and trabecular specification defects in B1KO suggest that β1 integrins may regulate asymmetric cell division. We wished to examine the distribution of mRNAs in the dividing cells at telophase or the two daughter cells when cytokinesis was completed, but the mid-body was still present.^7^ E8.75 heart sections were stained with acetylated α-tubulin and p120 and hybridized with probes to *Bmp10* or *Hey2* mRNA using RNAScope. We counted the number of mRNA signal dots in dividing cells and quantified the ratio between the two domains of the dividing cell at telophase or the two daughter cells after division. *Hey2* mRNAs display asymmetric distribution between daughter cells resulting from perpendicular divisions as the ratios of *Hey2* in luminal daughter to abluminal daughter were close to 0.5 (*Figure 5G, G1,* and *K*), but not between the daughter cells resulting from parallel division (*Figure 5I, I1,* and *L*). This suggested the different geometric locations or the different signals the two daughter cells received caused asymmetric levels of mRNAs. This phenomenon is an extrinsic asymmetric cell division and has been well-documented previously in *Drosophila* ovarian stem cells.^49^ In B1KO hearts, *Hey2* did not display an asymmetric distribution between the two daughter cells resulting from perpendicular or parallel cell division (*Figure 5H, H1, J, J1, K,* and *L*). We found *Bmp10* was asymmetrically distributed between the two daughter cells resulting from extrinsic asymmetric cell division, with the ratios of *Bmp10* in luminal daughter to abluminal daughter being close to 2 (*Figure 5M, M1,* and *Q*). In parallel divisions, the asymmetric distribution of *Bmp10* was not observed (*Figure 5N, N1,* and *R*). In B1KO hearts, *Bmp10* did not display an asymmetric distribution between the two daughter cells resulting from either perpendicular or parallel cell division (*Figure 5O, O1, P, P1, Q,* and *R*). Therefore, sister cells in all OCD display an equal amount of *Hey2* or *Bmp10* in B1KO, suggesting a failed asymmetric cell division (*Figure 5*). *Nkx2.5^Cre/+^; Itgb1^fl/fl^; mTmG* hearts, in which cell boundary was marked by the membrane-localized GFP in B1KO or RFP in control were applied to confirm that the asymmetric distribution of *Hey2* and *Bmp10* in B1KO was affected in B1KO hearts (see Supplementary material online, *Figure S6H*, *H1, I*, and *I1*). The failed asymmetric distribution of *Hey2* and *Bmp10* in OCD is consistent with the even distribution of *Hey2* and *Bmp10* in the trabecular and compact zones in B1KO (*Figure 2H, I, K,* and *L*).

### 3.7 B1KO hearts display a reduced Notch1 activation, but Notch1 reduction was not sufficient to cause cardiomyocyte organization defects

**Figure 6:**
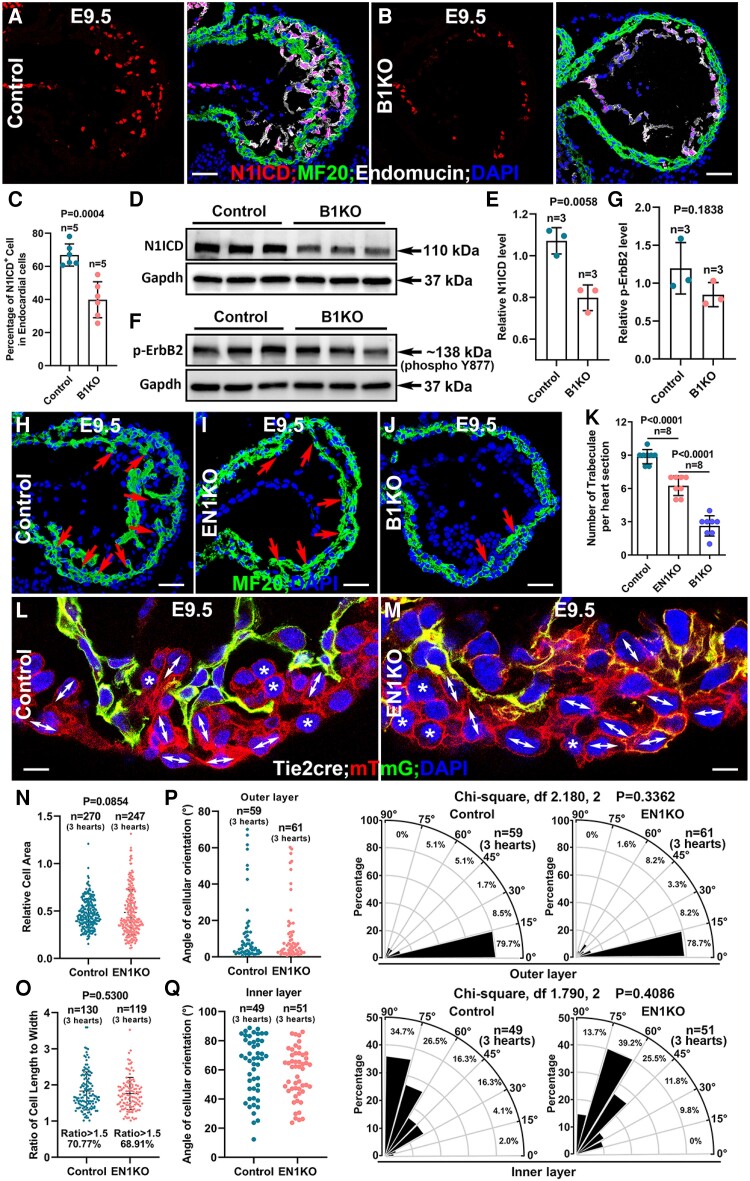
B1KO hearts display reduced Notch1 activation, but Notch1 reduction did not cause a cardiomyocyte organization defect. (*A–E*) Notch1 activation was reduced in the B1KO hearts, based on N1ICD IF staining (*A–C*, *n* = 5) and western blot (*D* and *E*, *n* = 3). (*F* and *G*) Nrg/ErbB signalling was not changed in the B1KO hearts, indicated by the unchanged protein level of its readout, p-ErbB2 (*n* = 3). (*H–K*) Endothelial-specific Notch1 knockout (EN1KO) hearts displayed trabecular growth defect, but the trabecular number per section of EN1KO heart is significantly greater than the B1KO hearts, indicating that the reduction of Notch1 activation is not the cause of the trabecular initiation defect in B1KO hearts (*n* = 8, eight sections from three hearts of each genotype). (*L–Q*) The cellular shape of cardiomyocytes was examined and quantified between the control and EN1KO heart, their cellular areas (*N*), percentage of oriented cells (*O*), and cellular orientation of both the inner and outer layer of the myocardium (*P*, *Q*) was not significantly different (cells for quantification were from three control and three B1KO hearts, respectively), and the percentages of cells with 0–30°, 30–60°, and 60–90° angle are compared via *χ*^2^ test. The statistic comparsion method in *C*, *E*, *G*, and *K* is two-tailed Student’s *t*-test. Scale bar: 50μm in (*A* and *M*), (*H*–*J*), 5μm in (*L* and *M*).

Multiple signalling pathways, including Notch1 and Nrg/ErbB, are required for trabecular morphogenesis.^50,51^ To examine if the Notch1 activation was affected in B1KO, Notch1 intracellular domain (N1ICD), a readout for Notch1 activation, was stained in sections from control and B1KO hearts. The percentage of N1ICD + endocardial cells, identified by Endomucin, in B1KO hearts, was significantly reduced compared to that of littermate control hearts (*Figure 6A–C*). The reduced Notch1 activation was confirmed by Western blot using the whole ventricles of control and B1KO hearts at E9.5 (*Figure 6D, E* and Supplementary material online, *Figure S7*). pErbB2, a readout for Nrg/ErbB signalling, was not reduced based on pErbB2 western blot using the whole ventricle at E9.5 (*Figure 6F* and *G* and Supplementary material online, *Figure S7*).

To directly test whether N1ICD reduction is sufficient to cause trabecular initiation defects and cellular organization defects observed in B1KO hearts, *Notch1* was deleted via *Tie2-Cre* to generate endothelial-specific Notch1 knockout (EN1KO), and the trabecular initiation and cellular organization were examined. We found that the EN1KO hearts displayed trabeculation defects and died before E10.5, but the number of trabeculae per section in EN1KO was significantly more than the B1KO (*Figure 6H–K*). The cellular areas and cellular orientation patterns in the inner and outer layer of the myocardium were not significantly different between the control and EN1KO (*Figure 6 L–Q*). This suggested cellular orientation and organization were not affected in the EN1KO hearts, and the reduced Notch1 activation was not responsible for the cellular organization and distribution defects in B1KO.

### 3.8 β1 integrins regulate cellular behaviour and organization autonomously

**Figure 7:**
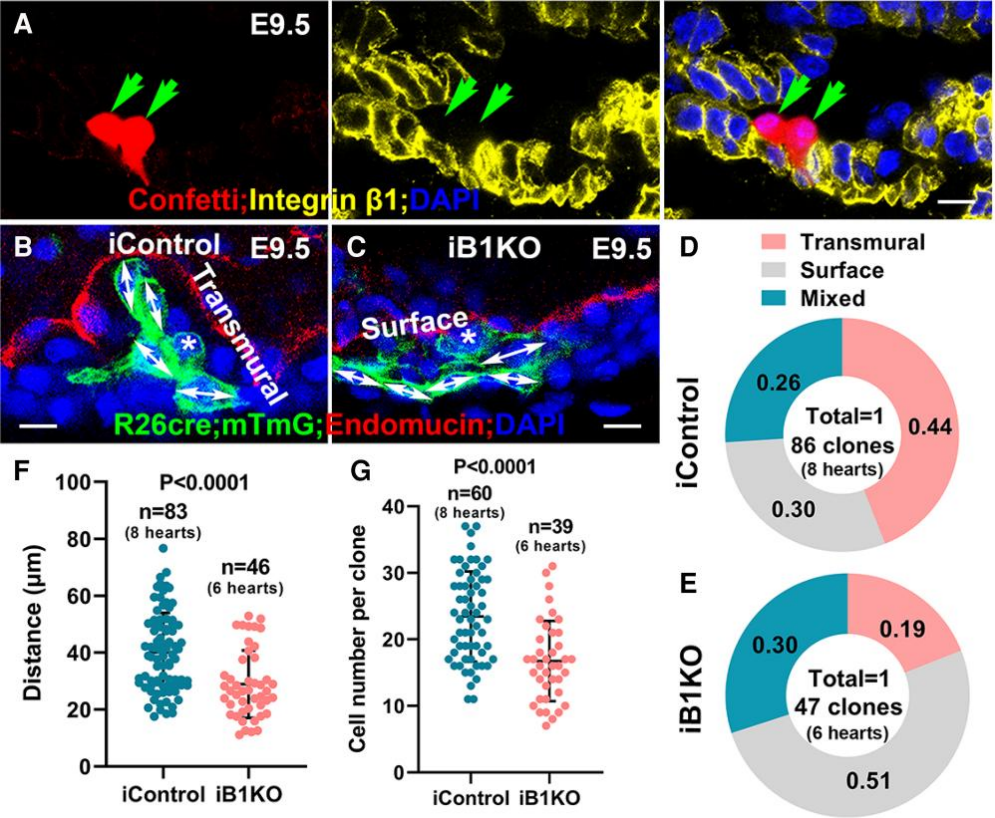
Single-cell lineage tracing shows abnormal cellular behaviours. (*A*) β1 expression was not detected in E9.5 iB1KO clones. (*B–E*) E9.5 iControl hearts showed a significantly higher percentage of transmural clones but a lower percentage of surface clones than iB1KO hearts (*n* = 6–8). (*F*) iB1KO clones showed significantly shorter migration distances compared with iControl clones. (*G*) iB1KO clones displayed a smaller clone size compared with iControl via Mann–Whitney test (clones for quantification were from eight icontrol and six iB1KO hearts, respectively). Scale bar: 10 μm.

We applied a mosaic model to determine if the effects of *Itgb1* deletion on cellular organization and behaviours are cell autonomous. We employed the inducible sparse lineage tracing system coupled with whole embryo clearing to genetically label, trace, image, and analyse individual clones at single cell levels.^29^ The improved imaging depth and scale of the cleared hearts allow for comprehensive 3D reconstruction of the heart and analysis of a single clone with spatial detail at the whole-heart scale. This approach enabled us to infer the division patterns and cellular behaviours during trabecular morphogenesis.^7,52^ Specifically, we crossed *ROSA26^CreERT2^* (iCre); *Itgb1^fl/+^*, in which iCre nuclear localization was induced by tamoxifen,^28^ with the reporter female mouse *ROSA26-Confetti* (*Conf*); *Itgb1^fl/fl^* or *mTmG*; *Itgb1^fl/fl^*. mTmG reporter was only used to determine the cell shape and cell size. *Conf* reporter mice can stochastically generate nuclear green, cytoplasmic yellow, cytoplasmic red, or membrane-bound blue cells upon Cre-mediated recombination.^7,29^ Tamoxifen at a concentration of 20-μg/gram-body weight was delivered to pregnant females via gavage when embryos were at E7.75, a stage when the myocardium is a monolayer and the trabeculae are not yet initiated. Seventy-two hours after induction, single labelled cells have undergone several rounds of cell division to exhibit specific geometric patterns.^7^ Based on the geometric distribution and anatomical annotation of each clone, the clones were categorized into three different patterns as previously reported^7^: (i) surface or parallel clones, in which most cells localize to the outer layer of the myocardium and likely are derived from parallel division; (ii) transmural or perpendicular clones, in which the cells localize to both compact and trabecular zones with only one or two cells remaining in the outer compact zone and likely are derived from perpendicular division; (iii) other unclassified clones, in which all the cells of the clone localize to the trabecular zone and likely are derived from directional migration of the labelled cell. The extent of *Itgb1* deletion in *ROSA26^Cre/+^*; *Itgb1^fl/fl^* (iKO) was determined by immunostaining for β1 (*Figure 7A*). About 85% of the iKO clones (*n* = 25) showed the absence of Itgb1 (*Figure 7A*). We compared the clonal patterns between control (*iCre; Itgb1^fl/+^; mTmG)* and iKO and found that 44% of the control clones are perpendicular (*Figure 7B*), and 51% of the iKO clones are parallel (*Figure 7C*). The clonal patterns between control and iKO clones were significantly different based on a *χ*^2^ test (*Figure 7D* and *E*). iKO clones displayed a higher percentage of surface clones and lower percentages of transmural and trabecular clones, suggesting that the β1 integrins have roles in OCD and directional migration. We also examined the migration depth via the whole heart clearing and 3D imaging.^7^ The migration depth is defined by the distance from the innermost cell of the clone to the myocardial surface. We found that the iKO clones migrated a shorter distance compared to the control clones (*Figure 7F*). We also measured the number of cells in each clone and found that the number of cells of the control clones was significantly larger than the iKO clones (*Figure 7G*). These data suggested that β1 integrins regulated cardiomyocyte migration and proliferation in an autonomous manner during trabecular morphogenesis.

## 4. Discussion

Our study demonstrates that β1 is asymmetrically distributed and *Itgb1* deletion at an early stage of ventricular wall formation results in distinct cardiac phenotypes from those reported in previous studies in which *Itgb1* was deleted at later stages.^20,53–57^ Our study shows that β1 integrins are required at the early stage for cardiomyocytes to attach to the ECM network and that this engagement provides structural support for cardiomyocytes to maintain proper cell shape and normal cellular behaviours that establish proper cellular organization for trabecular formation. Deletion of *Itgb1* prevents cardiomyocytes from engaging the ECM network, causing abnormal cellular organization and failed trabeculation.

### 4.1 β1 integrins regulate heart formation and function in a spatiotemporal manner.

Integrins are transmembrane heterodimeric proteins and reside at the interface of cardiomyocytes and ECM. These molecules are critical to mediate the cell–cell and ECM interactions that allow for contraction.^32^*Itgb1*, *Itga6,* and *Itga5* are the three most highly expressed integrin subunit genes in the E9.5 heart based on mRNA deep sequencing (see Supplementary material online, *Table S1*). *Itgb1* is expressed in all types of cells in the heart, and global deletion of *Itgb1* is lethal at the pre-implantation stage.^15,16^ Endothelial-specific knockout of *Itgb1* via *Tie2-Cre* results in severe vascular defects and lethality at E10.5,^18,19^ and deletion of *Itgb1* via the inducible *VE-Cad^CreERT2^* disrupts endothelial cell polarity and arteriolar lumen formation.^17^ Cardiomyocyte-specific knockout of the *Itgb1* via *Mlc-2vCre* results in myocardial fibrosis and cardiac failure in the adult heart,^20^*Itgb1* deletion via *cTnT-Cre* or *Xmlc2^Cre^* results in defects in myocardial integrity and death at about E14.5,^21,22^ and *Itgb1* deletion via the transgenic *Nkx2.5Cre* line perturbs the trabecular compaction and cardiomyocyte proliferation with progressive cardiac abnormalities seen toward birth.^23^ In our study, *Itgb1* deletion via the knockin *Nkx2.5^Cre/+^* or *Nkx2.5^IRES-Cre/+^*, which is expressed in the heart earlier than the transgenic *Nkx2.5Cre* (possibly due to the promoter region of the transgenic *Nkx2.5Cre* not including all the regulatory elements), causes defects in trabecular formation. *Itga6* is enriched in the early heart as evidenced by *in situ* hybridization and immunostaining,^58^ and the specific expression of α6 in the trabecular zone during compaction^59^ suggests its potential functions in compaction. *Itga6* global knockout dies around birth due to epidermolysis bullosa, and cardiovascular abnormalities were not extensively examined.^60^ The *Itga5* global knockouts display a thicker compact zone, as do *Fn1* global knockouts,^61^ suggesting a cellular organization defect. Mice homozygous for a null mutation of the *Itgb5, Itga1,* or *Itgb3* develop, grow, and reproduce normally.^62–65^ The low expression of *Itgav* in the heart is maintained throughout cardiac development.^66^*Itgav* and *Itgb3* integrins could not functionally compensate for the loss of *Itgb1* function during specialization and terminal differentiation of cardiomyocytes.^67^*Itga4* deletion causes a defect in epicardial and coronary development.^68^*Itgb4* was not expressed in the heart.^58^ The B1KO hearts display a variety of defects in trabecular formation, specification, compaction, cellular orientation, cellular organization, and proliferation, suggesting that β1 integrins play multiple functions during trabecular morphogenesis. Our results indicate that β1 integrins are essential for ECM organization and extracellular space formation at early stages, and for cardiomyocytes attaching to the ECM. Our data suggest that β1-mediated cell–ECM interaction at an early stage is required to establish cell shape, cellular organization, and tissue architecture, while at a later stage, these functions may not be essential, as the cell shape, cellular organization, and tissue architecture are already established.

### 4.2 β1 integrins regulate cellular behaviours and organization during ventricular wall formation

It is worth noting that β1 was asymmetrically distributed in the myocardium and the individual cardiomyocytes, with the β1 subunit being enriched along the luminal side of the myocardium and cardiomyocytes at E9.5. Fn, a ligand for α5β1 integrins, is also asymmetrically distributed in the myocardium and cardiomyocytes (see Supplementary material online, *Figure S8A*), and it surrounds cardiomyocytes, potentially creating an Fn network for cardiomyocytes. Laminin 411 and collagen IV, two ligands for β1 integrins, are asymmetrically distributed to the luminal side of the myocardium and cardiomyocytes. Furthermore, the ablation of β1 interferes with Fn localization and stability (*Figure 1F* and Supplementary material online, *Figure S8B*), suggesting that multiple but distinct integrin–ECM interactions are essential for early cardiac morphogenesis. The asymmetric distributions of β1, Fn, laminin 411, and Collagen IV to the luminal side of the myocardium suggest a polarized myocardium, and this polarized ECM might establish a polarized network frame. The cardiomyocytes attach to the ECM scaffold in the myocardium via integrins. The ECMs are enriched with growth factors, provide growth cues for the cells inside, and provide the frame for the cardiomyocytes to be stabilized in the myocardium. Cardiomyocyte β1 integrins allow the cardiomyocytes to adhere to the ECM-network frame (see Supplementary material online, *Figure S8C*). This ECM-scaffold provides a supportive niche for the cellular organization, cellular behaviour, cardiomyocyte maturation, and ventricular wall specification. The disruption of the interaction between ECMs and cardiomyocytes by deleting *Itgb1* would prevent cardiomyocytes from engaging the ECM scaffold frame, such that the cardiomyocytes would lose their position in the myocardium, their normal cellular behaviours became awry, their cellular organization was disrupted, and the cells fail to initiate and establish the necessary tissue architecture to form trabeculae. The random cellular organization and impaired cell growth direction may cause abnormal distribution of cardiomyocytes between the trabecular and compact zone, resulting in reduced trabeculae and a thicker compact zone. Indeed, the data suggest that β1 integrins are required for cardiomyocyte organization and cellular distribution between trabecular and compact zones. The loss of β1 and separation of cardiomyocytes from the ECM likely disrupt the niche that fosters the extrinsic cell division and ventricular wall specification. However, the disruption of cell–ECM interaction in B1KO does not mean the cardiomyocytes are floating in the myocardium as N-Cadherin protein levels and localization in B1KO mice were unchanged and cell–cell adherens junctions appeared unaffected as cardiomyocytes remained linked to each other in the myocardium based on immunostaining and EM pictures. The Fn knockout further supports the model (see Supplementary material online, *Figure S8*), as global Fn knockout hearts display a myocardial organization defect with an absence of lumen, and Fn is required for heart morphogenesis.^35^

In summary, the ECM-network provides geometrical confinement and a supporting frame for the cardiomyocytes inside the myocardium. The geometrical confinement and supporting frame appear to be critical for the regulation of proper cardiomyocyte shape, cellular behaviour, and organization. β1 integrins play a major role in establishing cell–ECM interaction, which is required to modulate the cell shape in response to signalling and physiological cues to achieve proper cellular behaviour (see Supplementary material online, *Figure S8*). This cell–ECM interaction is required to establish the cardiomyocyte organization or arrangement inside the developing myocardium. The perturbation of β1 integrin disrupts the correct geometrical confinement and supporting structure, resulting in trabeculation defects.

**Translational perspective**

Congenital heart defects are cardiac structural defects. This study reveals the intricate interplay between cardiomyocytes and their extracellular matrix mediated by β1 integrins is fundamental in organizing the cardiomyocytes and shaping the structure and function of the developing heart. This study illuminates the pivotal role of the asymmetrically localized β1 integrins in orchestrating cardiomyocyte behaviour and organization during ventricular wall morphogenesis in mice. Further exploration of these molecular pathways and their manipulation could pave the way for novel therapeutic interventions in congenital heart diseases, offering hope for improved clinical outcomes in affected individuals.

## Supplementary Material

cvae111_Supplementary_Data

## Data Availability

All data are available in the main text or the Supplementary material online. Unpublished data are available upon request. The mouse lines that we generated are available upon request.
