# Supplementary material for: β1 integrins regulate cellular behaviour and cardiomyocyte organization during ventricular wall formation
Source: Cardiovasc Res. 2024 May 24;120(11):1279–94. doi: 10.1093/cvr/cvae111 (PMC11416060; doi:10.1093/cvr/cvae111)
Supplement: cvae111_Supplementary_Data [file cvae111_supplementary_data.pdf]

CVR-2023-1092

β1 integrins regulate cellular behavior and cardiomyocyte organization during ventricular wall formation.

Lianjie Miao[1], Yangyang Lu[1], Anika Nusrat[1], Luqi Zhao[1], Micah Castillo[2], Yongqi Xiao[1], Hongyang Guo[1], Yu Liu[1], Preethi Gunaratne[2], Robert, J Schwartz[2], Alan R Burns[3], Ashok Kumar[1], C. Michael DiPersio[4], and Mingfu Wu[1*]

Running Title: β1 integrins are required for ventricular wall formation.

[1]Pharmacological and Pharmaceutical Sciences, College of Pharmacy, University of Houston, Houston, TX 77204-5039
[2]Department of Biology and Biochemistry, University of Houston Sequencing and Gene Editing Core, University of Houston, Houston, TX.
[3]College of Optometry, University of Houston, Houston, TX.
[4]Department of Surgery, Albany Medical College, Albany, NY 12208

*Correspondence to Mingfu Wu, Ph.D.

Department of Pharmacological and Pharmaceutical Sciences,
College of Pharmacy, University of Houston,
Houston, TX 77204-5039
*Correspondence: mwu25@central.uh.edu

Phone: (713)743-9880

Fax: (713)743-1884

Subject codes: Trabecular morphogenesis, ventricular wall specification, integrin-extracellular matrix interaction, cellular organization, cell behavior.

Category: Original Articles

Supplemental Materials and Methods

Immunofluorescence (IF)

Immunofluorescence (IF) staining was performed as previously described[1, 2]. Briefly, embryos or heart samples were fixed in 4% PFA for 2 hours at room temperature or overnight at 4°C. After fixation, samples were washed with PBS, embedded in OCT, frozen at -80°C and sectioned at 10 µm per section. Sections were collected on glass slides and immersed in PBS for 10 min to remove OCT. The sections were then permeabilized with PBT (0.5% Tween in PBS) (if needed) and then blocked for 30 min with TNB blocking buffer (Perkin Elmer, FP1020, Waltham, MA, USA) at RT. After blocking, the sections were incubated with primary antibodies diluted in blocking buffer overnight at 4 ◦C. Then, the slides were washed with PBT 3 × 10 min at RT, followed by secondary antibodies incubation at RT for 1 hour. After secondary antibody incubation, the sections were counterstained with DAPI and mounted in mounting medium (Vectashield, H-1000-10) for confocal imaging. Primary antibodies were used against the following targets: Endomucin (1:100, Santa Cruz, sc-65495), MF20 (1:100, DSHB), PECAM (1:50; BD Pharmingen, 550274), N1ICD (1:50; Cell Signaling, 4147S), P57 (1:200, Abcam, ab75974), Integrin β1 (1:100, Millipore, MAB1997), Integrin a5 (1:100, Thermo, PA5-79529), Integrin a6 (1:100, Thermo, 14-0495-85), Fibronectin (gift from Dr. Paula J. Mckeown-Longo's lab), Laminin (gift from Dr. Susan Laflamme lab), Collagen IV (1:500, Sigma Aldrich, AB756P), P21 (1:100, Abcam, ab109199), p-Smad 1/5/8 (1:500, Cell Signaling, 9511S), Irx3 (1:100, Santa Cruz, sc-30157), P120 (1:100, Santa Cruz, sc-1101), Acetylated tubulin (1:400, Sigma Aldrich, T6793), N-cadherin (1:200, BD Pharmingen, 610920), HABP (1:100, Millipore, 385911), Versican (1:200, Thermo, PA1-1748A), and Cleaved Caspase-3 (1:400, Cell Signaling, 9661S).

Cardiomyocyte proliferation assay by BrdU pulse labeling

Details can be found in our published protocol[3]. Briefly, pregnant females were intraperitoneally injected with BrdU for 1 hour before embryo harvesting. MF20 or cTnT was stained to identify cardiomyocytes. Proliferation index was calculated by dividing the number of BrdU and cTnT double positive cells by the number of DAPI stained cTnT positive cells in trabecular or compact myocardium and multiplying by 100. A minimum of six sections per heart from at least three hearts for each genotype was quantified at each stage.

Whole embryo immunofluorescence staining and clearing

Whole embryos were stained as previously described[2]. Briefly, whole embryos were fixed for 2-4 hours in 4% paraformaldehyde, permeabilized for 2 hours in PBS-Tween 20, blocked with 3% BSA, and then incubated with the primary antibody for ~24 hours. After three washes, the embryos were incubated with the secondary antibody for 24 hours, followed by three more washes with PBS. After staining, embryos were cleared using RapiClear® 1.52 (SunJin Lab, RC152001).

Single mRNA molecule in situ hybridization (ISH)

Single mRNA molecule in situ hybridization (ISH) and immunofluorescent staining (IFS) were performed using the RNAscope 2.5 HD (RED) Assay (Advanced Cell Diagnostics, 322360) according to the manufacturer's instructions and our published protocol[4-6], which enables the detection of single mRNA molecules. Briefly, after fixation for 24 hours, the embryos were embedded in OCT, frozen and sectioned. The mRNA expression level in each cell was determined based on the number of mRNA molecules or signal intensity using the confocal scanned pictures, and at least three sections for each cell were quantified.

Imaging

Zeiss LSM 880-NLO confocal microscope system with an Airyscan detector and STED Nanoscopes Leica TCS SP8 STED were used for confocal imaging and whole mount imaging in

a z-stack manner. Stereo images of the heart or embryos were harvested using a stereoscope (Leica M205 FA).

Quantitative evaluation of the orientation of the cardiomyocyte division plane

Orientation of the cardiomyocyte division plane was determined by imaging heart sections or cleared whole hearts (described below). Sections or whole hearts of *Nkx2.5*$^{Cre/+}$*; Itgb1*$^{fl/fl}$*; mTmG* and *Nkx2.5*$^{Cre/+}$*; Itgb1*$^{fl/+}$*; mTmG* at E9.25 were stained with acetylated α-tubulin to determine the spindle orientation. Z-stack images were acquired using a STED Nanoscopes Leica TCS SP8 STED equipped with multi-photon excitation at 1-3 µm per section. Spindle orientations of left ventricular cardiomyocytes in anaphase or early telophase where both centrosomes and nuclei were in the same focal plane were quantified. The spindle orientation was determined by the angle between the spindle axis, determined by the two centrosomes, and the basement membrane or heart surface reference line. Division planes positioned at 60-90° to the basement membrane were classified as perpendicular, those oriented at 0-30° were classified as parallel, and those oriented between 30-60° were considered non-classified. The data shown are a combination of separate analyses from two investigators.

Quantitative evaluation of the orientation of cardiomyocytes

Orientation of the cardiomyocyte plane was determined by imaging heart sections or cleared whole hearts. Cardiomyocytes of *Nkx2.5*$^{Cre/+}$*; Itgb1*$^{fl/fl}$*; mTmG* and *Nkx2.5*$^{Cre/+}$*; Itgb1*$^{fl/+}$*; mTmG* heart at E8.75 were labeled with membrane GFP, and whether a cell is oriented can be determined by the length to width of the cell. If the ratio of length to width of a cell is greater than 1.5, then this cell is oriented[7]. The cellular orientation was determined by the angle between the longitudinal axis of an oriented cell to the heart surface reference line. Cell orientations with 60-90° angles are classified as perpendicular, those oriented at 0-30° are classified as parallel, and those oriented between 30-60° are considered non-classified. The data shown are a combination of separate analyses from at least three hearts. Cellular orientations of cardiomyocytes of the outer curvature region of hearts from the same litter were compared.

Western Blot Analysis

Western blot was performed as previously described[1, 8]. Briefly, E9.5 hearts were harvested and lysed in RIPA buffer. Protein concentration was determined using the BCA kit (Thermo Fisher, 23225), and equal amounts were run on SDS-PAGE using 4–20% Mini-PROTEAN® TGX™ Precast Protein Gels (Bio-Rad, 4561093) and transferred onto PVDF membranes (GE Healthcare Life Science, 10600023) following standard protocols. The antibodies used in the study include GAPDH (1:1000, Santa Cruz, sc-25778), N1ICD (1:1000; Cell Signaling, 4147S), N-cadherin (1:1000, BD Pharmingen, 610920), p-ErbB2 (1:1000, abcam, ab47262), HABP (1:1000, Millipore, 385911), and Versican (1:1000, Thermo, PA1-1748A).

Inducible lineage tracing and mosaic analysis

Heterozygous *Rosa*$^{CreERT2/+}$ males were crossed with homozygous *mTmG* or *Confetti* females to produce reporter embryos. Tamoxifen (T5648, Sigma), dissolved in sunflower seed oil (S5007, Sigma), was gavaged to pregnant females 7.75 days after coitus or at a specified time, with a dose of tamoxifen at 100 or 50 µg per gram body weight. The embryos were harvested at the indicated age and used for IFS or whole-mount staining. The clones were imaged and then analyzed for cell count and distance (z) from the heart surface to the innermost cell of the clone.

mRNA deep sequencing

Total RNA was isolated from nine E9.5 hearts from both control and B1KO for each experiment. As an indication of quality, the RNA had an integrity number of 8 or greater by Bioanalyzer (Agilent Technology). Samples for mRNA deep sequencing were prepared according to the manufacturer's protocol (mRNASeq 8-Sample Prep Kit, Illumina). The samples were sequenced by the Microarray Core Facility at the University of Texas, Southwestern Medical Center at Dallas.

A HiSeq 2000 system (Illumina) was used for SE-50 sequencing (single-ended 50 bp reads), with over 30×10^6 'reads' per sample. Basic data analysis was performed with CLC-Biosystems Genomic Workbench analysis programs to generate quantitative data for all genes. The quality filtered and trimmed reads were aligned to an annotated mouse reference genome downloaded from the Ensembl Genome Browser. cDNA fragments were mapped back to individual transcripts. After normalization, the RNA-Seq fragment count was used to measure the relative abundance of transcripts. Basemean is the average of the normalized count values, divided by size factors, taken over all samples, including the control and knockout samples per Illumina. Three independent experiments were performed. Relative expression levels of genes (ratio of B1KO to control) with $P<0.05$ were considered significantly different.

Electron microscopy imaging

Embryos at E9.5 were processed for serial block-face scanning electron microscopy (SBF-SEM). After isolating the embryos at the desired age, samples were kept in a fixative solution (0.1M sodium cacodylate buffer containing 2.5% glutaraldehyde and 2 mM calcium chloride) for 2 hours at room temperature while gently agitating, then kept at 4 degrees overnight. Then, the embryos were washed in a washing buffer (0.1 M sodium cacodylate buffer containing 2 mM calcium chloride) three times for 10 min each at room temperature. Fixed embryos were stained with heavy metals (Fe, OsO4, uranyl acetate, lead) before dehydration through an acetone series and embedment in Embed 812 resin (Electron Microscopy Sciences, USA) containing Ketjenblack EC600JD (Lion Specialty Chemicals Co., Japan). The resin-embedded blocks were sputter-coated with gold to further reduce charging during block-face imaging. Tissue blocks were serially sectioned at 100 nm steps using a Gatan 3View2 microtome (Gatan, USA) mounted to a Mira 3 scanning electron microscope (Tescan, USA). Back scatter electron (BSE) detection was used to image the block-face. Serial imaging was conducted under high vacuum (0.047 Pa) using a Schottky emitter and an accelerating voltage of 9.0 keV. Beam intensity ranged from 5-7 on a scale ranging from 1-20, with a pixel dwell time of 32 μs, and a spot size of 4-7 nm. Magnification ranged from 500-36,900x and pixel size from 7.32-542 nm. Subsequent three-dimensional segmentation and reconstruction were conducted using Amira 6.0.1 software (FEI Company, Hillsboro, OR).

Rodent CO_2 Euthanasia

The mouse is placed in an empty chamber with a CO_2 delivery lid. The CO_2 flow from the gas cylinder is initiated at a controlled rate, gradually displacing 30-70% of the chamber volume per minute. When the mouse has been rendered unconscious and is no longer breathing, turn off the CO_2. Observe and palpate that the mouse is lack of respiration and heartbeat. Then perform the cervical dislocation as a secondary technique to assure death of the mouse.

Statistics analysis

Data are shown as mean ± standard deviation. Two-tailed Student's t-test was used to analyze comparisons between two groups that display normal distribution assessed by Goodness of fit test, and Mann–Whitney test was used via the Prism 10 in case normal distribution could not be confirmed. Chi-square test was used to compare division patterns between control and knockout as specified. A P-value of 0.05 or less was considered statistically significant.

1. Zhao C, Guo H, Li J, Myint T, Pittman W, Yang L, Zhong W, Schwartz RJ, Schwarz JJ, Singer HA, Tallquist MD, Wu M. Numb family proteins are essential for cardiac morphogenesis and progenitor differentiation. *Development* 2014;**141**:281-295.
2. Shaikh Qureshi WM, Miao L, Shieh D, Li J, Lu Y, Hu S, Barroso M, Mazurkiewicz J, Wu M. Imaging Cleared Embryonic and Postnatal Hearts at Single-cell Resolution. *J Vis Exp* 2016.

3. Wu M, Smith CL, Hall JA, Lee I, Luby-Phelps K, Tallquist MD. Epicardial spindle orientation controls cell entry into the myocardium. *Dev Cell* 2010;**19**:114-125.
4. Li J, Miao L, Shieh D, Spiotto E, Li J, Zhou B, Paul A, Schwartz RJ, Firulli AB, Singer HA, Huang G, Wu M. Single-Cell Lineage Tracing Reveals that Oriented Cell Division Contributes to Trabecular Morphogenesis and Regional Specification. *Cell reports* 2016;**15**:158-170.
5. Miao L, Li J, Li J, Lu Y, Shieh D, Mazurkiewicz JE, Barroso M, Schwarz JJ, Xin HB, Singer HA, Vincent PA, Zhong W, Radice GL, Wan LQ, Fan ZC, Huang G, Wu M. Cardiomyocyte orientation modulated by the Numb family proteins-N-cadherin axis is essential for ventricular wall morphogenesis. *Proc Natl Acad Sci U S A* 2019;**116**:15560-15569.
6. Miao L, Li J, Li J, Tian X, Lu Y, Hu S, Shieh D, Kanai R, Zhou BY, Zhou B, Liu J, Firulli AB, Martin JF, Singer H, Zhou B, Xin H, Wu M. Notch signaling regulates Hey2 expression in a spatiotemporal dependent manner during cardiac morphogenesis and trabecular specification. *Sci Rep* 2018;**8**:2678.
7. Wyatt TP, Harris AR, Lam M, Cheng Q, Bellis J, Dimitracopoulos A, Kabla AJ, Charras GT, Baum B. Emergence of homeostatic epithelial packing and stress dissipation through divisions oriented along the long cell axis. *Proc Natl Acad Sci U S A* 2015;**112**:5726-5731.
8. Li J, Miao L, Zhao C, Shaikh Qureshi WM, Shieh D, Guo H, Lu Y, Hu S, Huang A, Zhang L, Cai CL, Wan LQ, Xin H, Vincent P, Singer HA, Zheng Y, Cleaver O, Fan ZC, Wu M. CDC42 is required for epicardial and pro-epicardial development by mediating FGF receptor trafficking to the plasma membrane. *Development* 2017;**144**:1635-1647.

Suppl. Figure 1

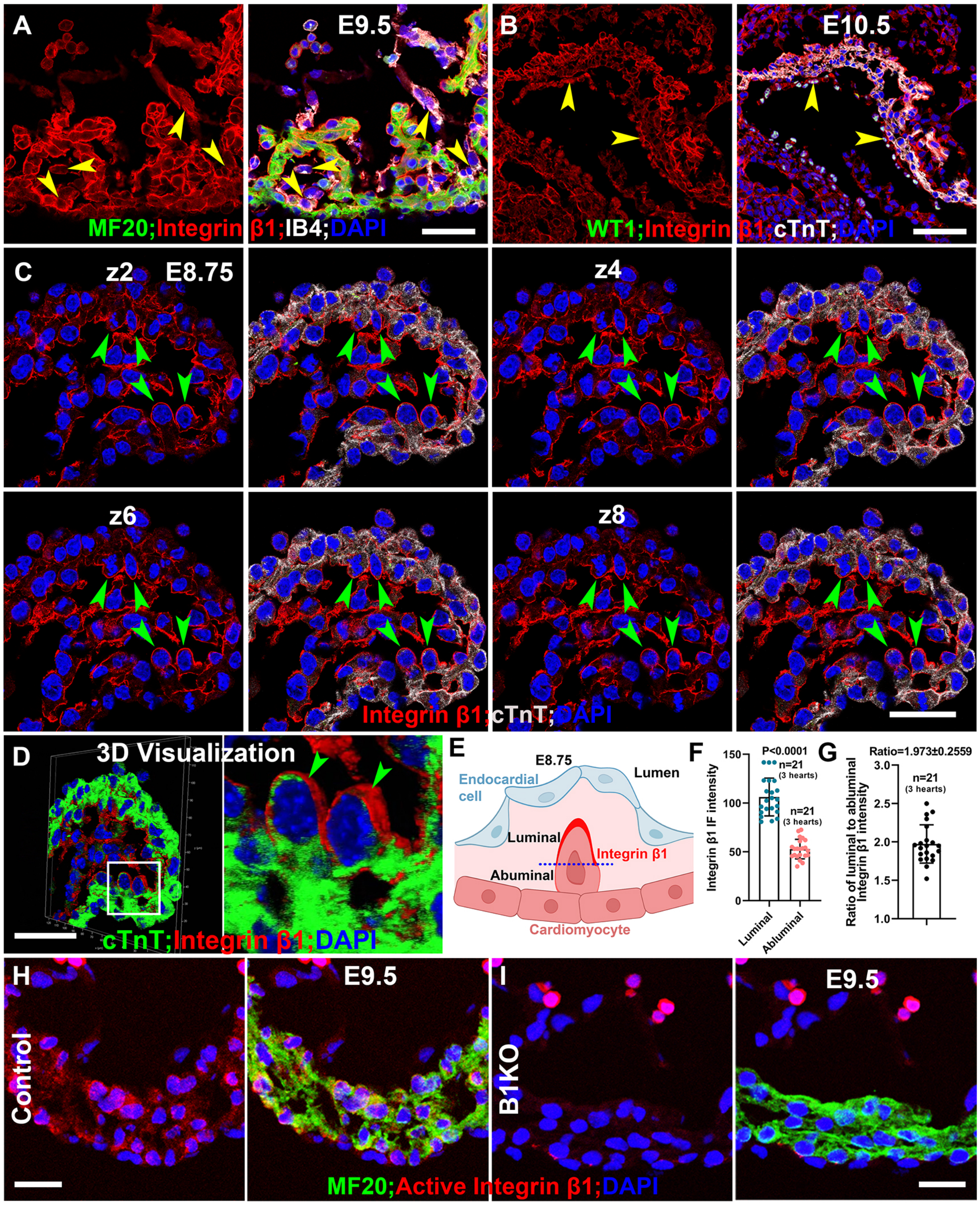

Suppl. Figure 2

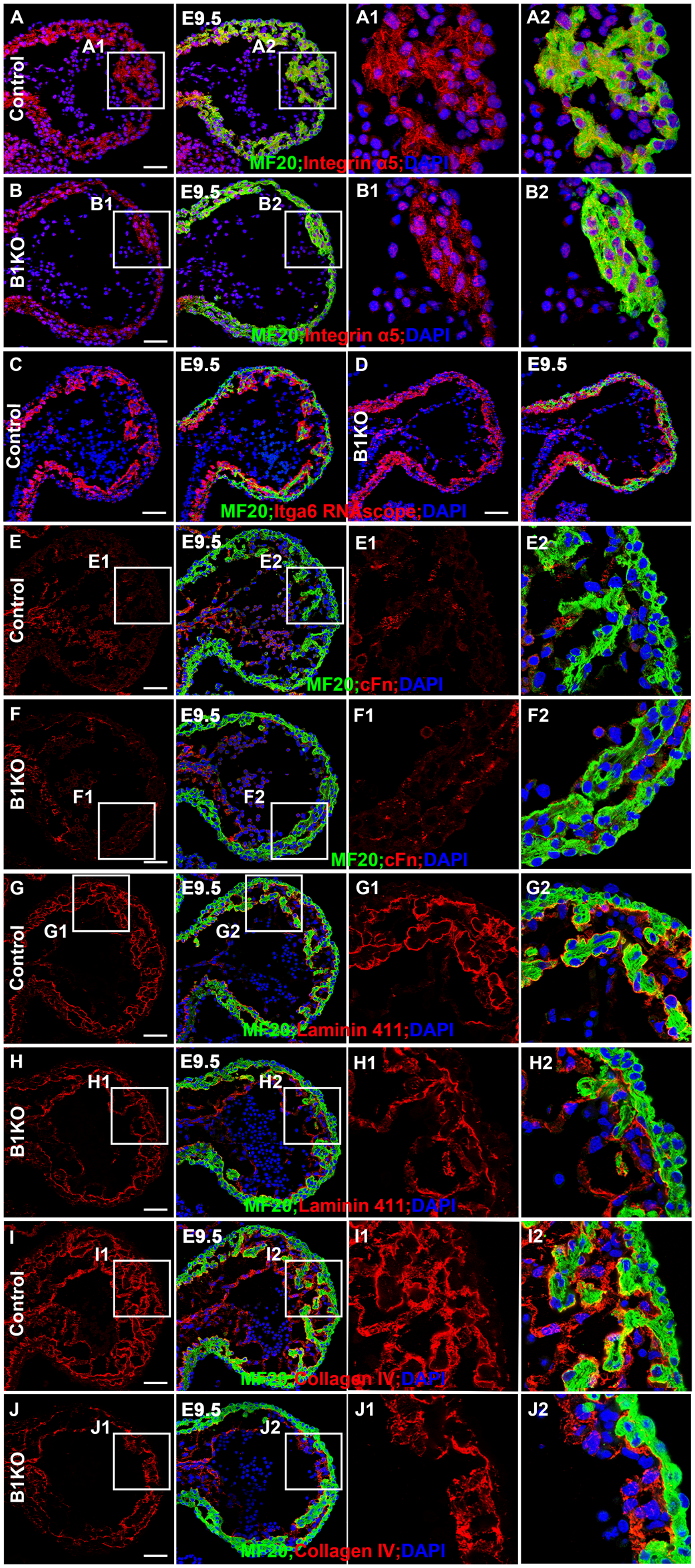

Suppl. Figure 3

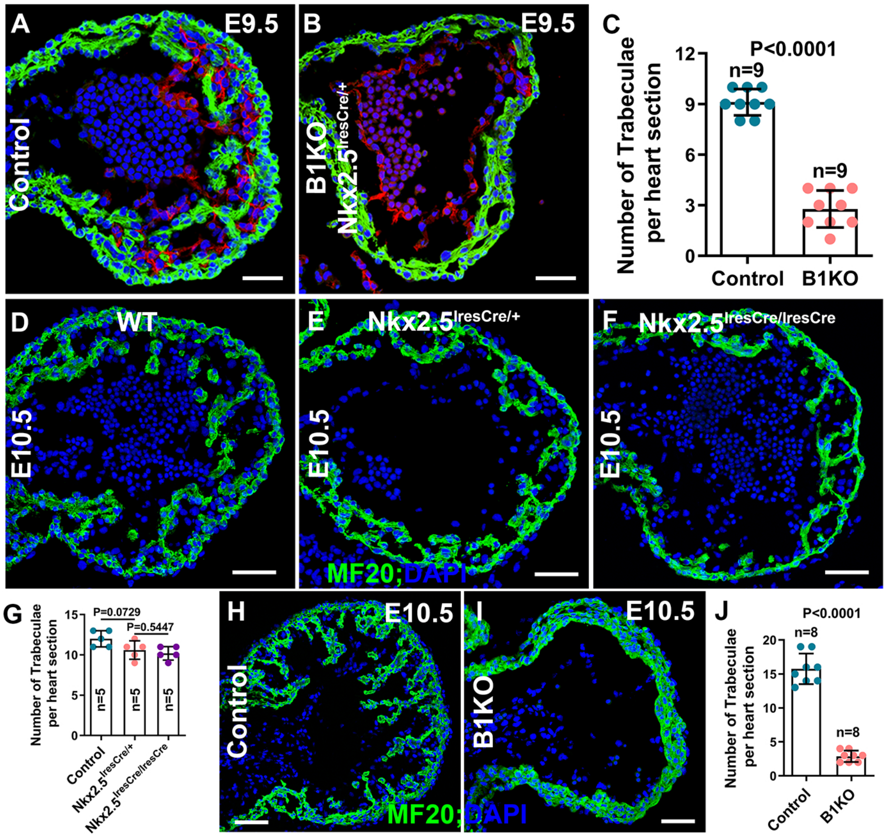

Suppl. Figure 4

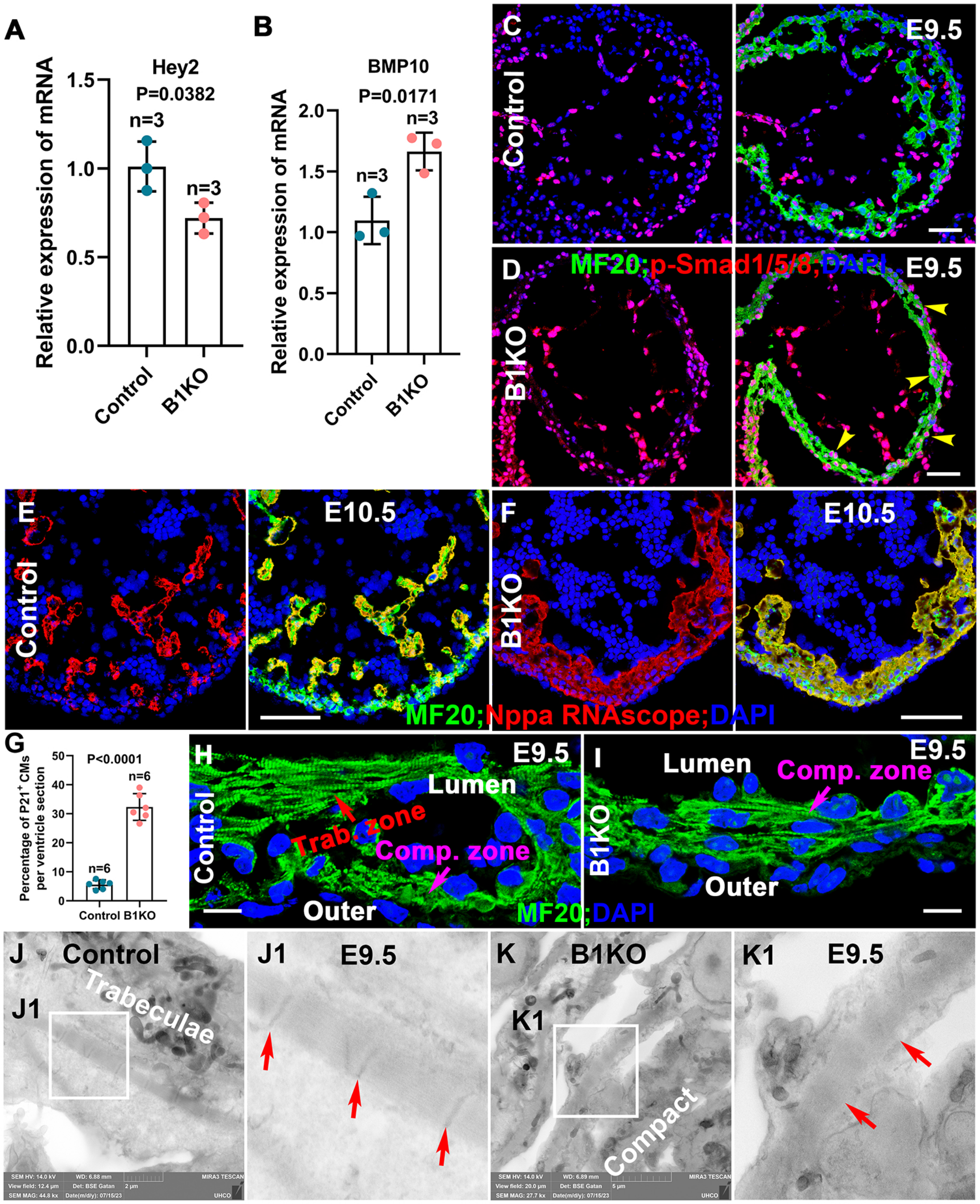

Suppl. Figure 5

Gel #1 for Figure 3C

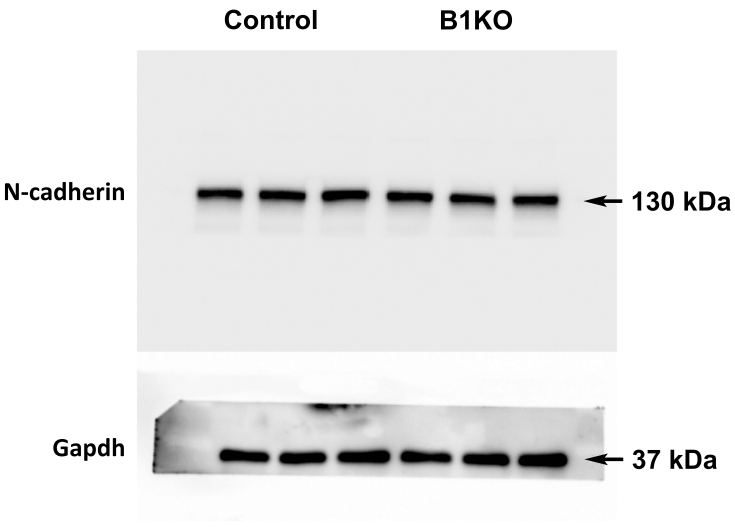

Gel #2 for Figure 3L

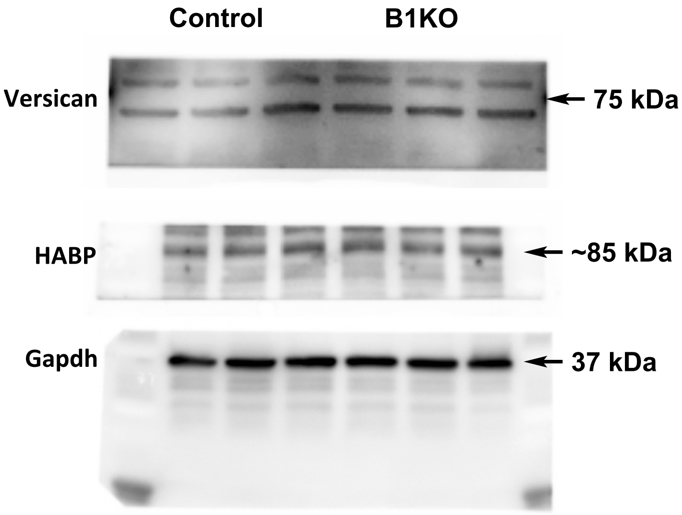

Suppl. Figure 6

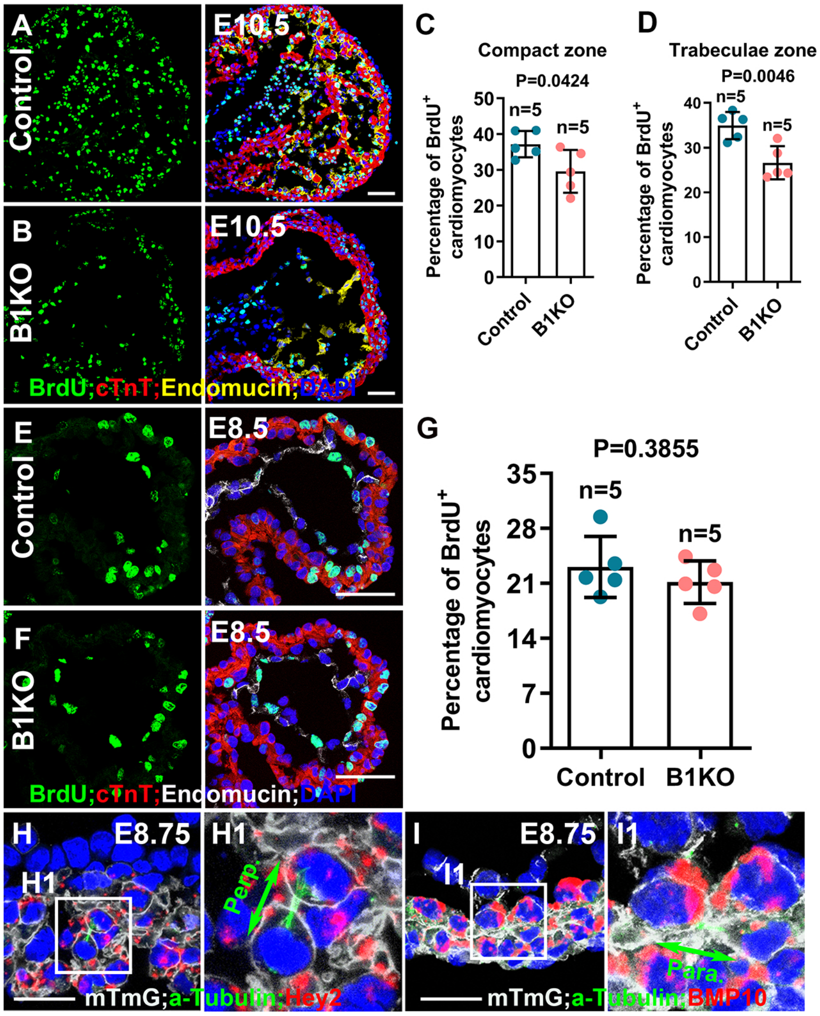

Suppl. Figure 7

Gel #1 for Figure 6D

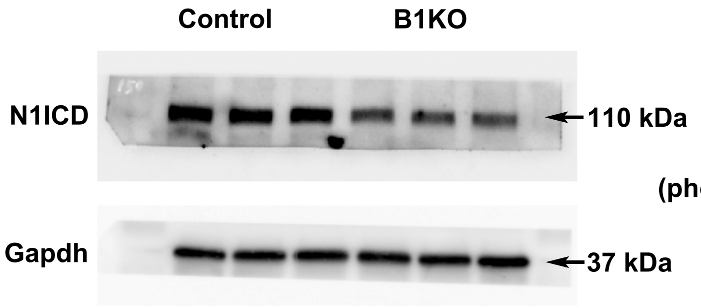

Gel #2 for Figure 6F

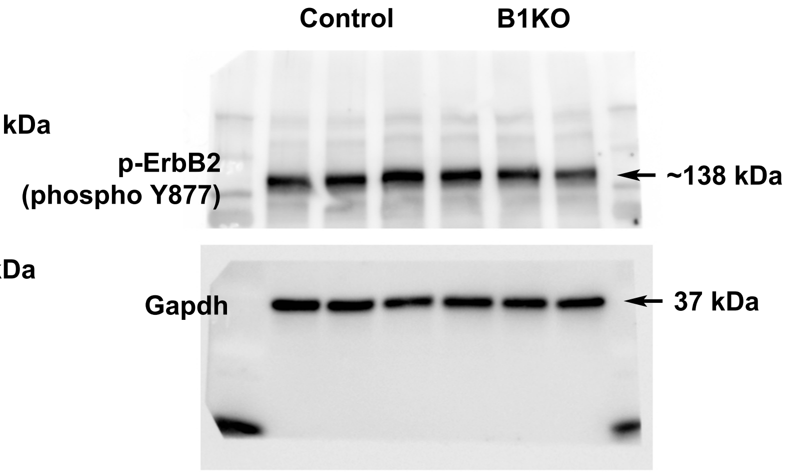

Suppl. Figure 8

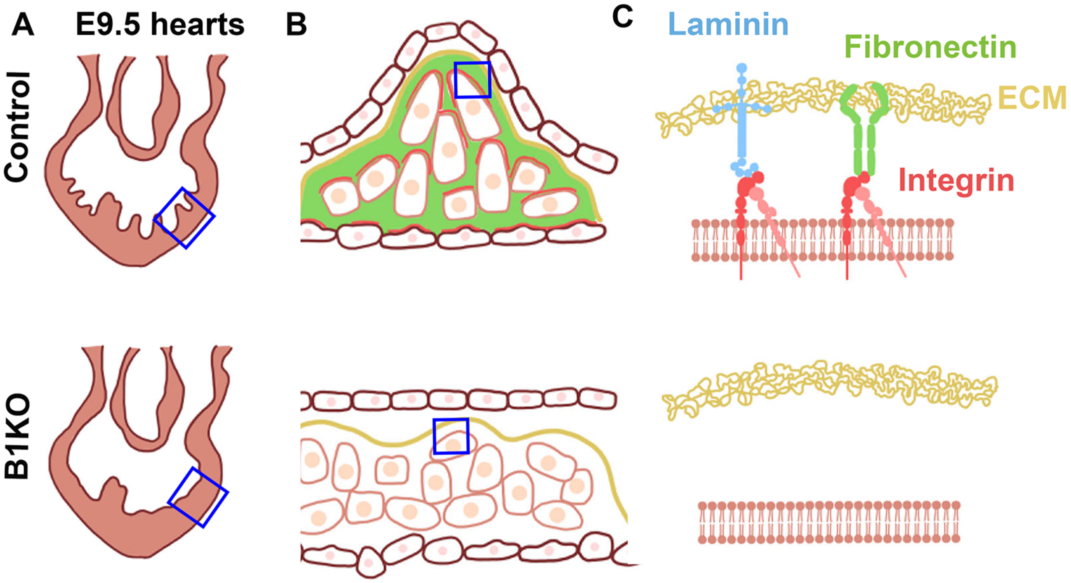

Tabel 1. Expressions of Integrins and their ligands in E9.5 hearts

symbol	baseMean	log2FoldChange	pvalue
Itga6	15496.92185	0.335664289	4.18E-10
Itga5	4807.254709	-0.061236291	0.20244954
Itgav	2142.962146	-0.038447686	0.707961797
Itga4	1876.231852	-0.252868185	0.000496303
Itga9	1824.099191	-0.046879344	0.53081939
Itga1	1275.069268	-0.245572751	0.002105139
Itga3	737.6016552	-0.150938337	0.138973659
Itga2b	430.4990634	0.126087859	0.495450734
Itga10	155.2980159	0.160179897	0.381722821
Itga8	141.1315812	-0.47723196	0.050037637
Itga2	61.24040399	0.021476907	0.942211481
Itga7	48.70622252	-0.832033757	0.046689928
Itgae	36.1021102	-0.017610151	0.961376462
Itgal	20.64407217	0.272856541	0.589842947
Itga11	20.61786085	-0.094726064	0.852970763
Itgad	14.23869144	0.102638633	0.863373361
Itgam	9.445725768	0.550455168	0.449605271
Itgax	1.067665895	-2.046504706	0.404620739
Itgb1	13097.54951	-0.806979211	3.20E-84
Itgb1bp2	1680.649481	0.059181393	0.390070033
Itgb3	1244.226623	-0.215286178	0.008090809
Itgb5	1234.860529	0.097639746	0.227267051
Itgb1bp1	343.9195277	-0.090082488	0.50564828
Itgb3bp	341.835156	-0.099062994	0.442618139
Itgb8	264.9667078	-0.093060695	0.522954196
Itgb2	38.12422731	0.367946672	0.35432961
Itgb4	26.39955801	0.228166267	0.66007866
Itgb7	4.529116186	1.173006406	0.289827753
Itgb6	2.100802743	0.346116136	0.825130566
Itgb2l	0.921997253	0.062760271	0.981310739
Lamc1	11137.66658	-0.008979321	0.836020299
Lamb1	8290.679242	0.058119707	0.219516928
Lama4	5624.804458	-0.002547953	0.955238015
Lama5	3268.083023	-0.17772571	0.016466224
Lamb2	3101.234381	0.200027277	0.036551497
Lama1	452.468331	-0.014017722	0.89969672

Lama2	301.5820561	0.05231657	0.697809472
Lamc2	145.9615107	-0.554639512	0.005402435
Lamb3	78.32032565	0.260448715	0.425521045
Lama3	41.49436877	-0.165902076	0.640995185
Lamc3	19.21278617	-0.937737738	0.122270688
Fn1	54378.72786	0.036682721	0.312994843
Vcam1	3191.127312	0.185906499	0.002174042
Vtn	6.65353464	-3.457998104	0.00614626
Col4a1	20866.09636	-0.031527466	0.465346995
Col2a1	14377.64281	-0.469676661	3.62E-10
Col18a1	13285.87809	-0.09264386	0.107736858
Col4a2	12524.17741	-0.031760744	0.531309141
Col3a1	5697.927173	-0.391217463	2.89E-06
Col4a5	5229.892031	-0.075922729	0.194680351
Col5a1	5048.677797	0.055519868	0.435512861
Col5a2	3061.962381	-0.103096736	0.055056056
Col1a2	1992.371686	0.061368255	0.363804442
Col4a6	1646.848477	-0.17155072	0.013045162

Table 2. Survival rate

Age	Total	KO	Harvested/Expected percentage of KO
E8.5	165	41	25/25
E9.5	483	105	22/25
E10.5	144	28	19/25
E11.5	25	1	4/25
E12.5	12	0	0/25

Suppl. Figure legends

Suppl. Figure 1. Asymmetrical distribution of Integrin β1.

(A) Immunostaining showed β1 expression in endocardial cells, identified by Isolectin B4 (IB4). (B) Immunostaining showed β1 expression in epicardial cells, identified by WT1. (C) β1 is asymmetrically distributed in dividing cardiomyocytes, with enrichment to the membrane of the luminal side. A 10μm-thick section was imaged via a z-stack manner with a 0.5μm interval. (D) Reconstructed 3D image showed the β1 distribution. (E-G) Quantification of the average intensity of β1 of luminal and abluminal side (n=21, 21 cells for quantification were from 3 control hearts), two-tailed Student's t-test. (H&I) The active β1 did not show obvious asymmetric distribution based on immunostaining. Scale bar: 10μm.

Suppl. Figure 2. Expression of β1 integrin ligands in the E9.5 hearts.

(A&B) Immunostaining for α5 integrin subunit showed that it is expressed in both trabecular and compact cardiomyocytes, and its expression pattern didn't change in the B1KO hearts compared with control hearts. (C&D) RNAscope revealed that *Itga6* mRNA level didn't significantly differ between control and B1KO hearts based on RNAscope. (E&F) The cellular isoform of Fibronectin (cFn) is mainly expressed in the AV canal but not in the ventricles by immunostaining, and its expression didn't change in the B1KO hearts. (G-J) Immunostaining for Laminin 411 and Collagen IV showed that they are expressed in the basement membrane and asymmetrically enriched to the luminal side of the myocardium, and their expression didn't change in the B1KO hearts. Scale bar: 50μm.

Suppl. Figure 3. *Nkx2.5$^{cre/+}$* and *Nkx2.5$^{IresCre/+}$* mediated Itgb1 deletion causes similar phenotypes.

(A-C) E9.5 B1KO hearts mediated by *Nkx2.5$^{IresCre/+}$* recapitulate the phenotypes of B1KO hearts mediated by *Nkx2.5$^{cre/+}$*, with a significantly smaller number of trabeculae per section and a thicker ventricular wall (n=9, 9 sections from 3 hearts of each genotype), two-tailed Student's t-test. (D-G) E9.5 embryos with *Nkx2.5$^{IresCre/+}$* or *Nkx2.5$^{IresCre/IresCre}$* did not show obvious trabeculation defect (n=5, 5 sections from 3 hearts of each genotype), two-tailed Student's t-test. (H-J) E10.5 B1KO hearts displayed trabeculation defect, with fewer trabeculae (n=8, 8 sections from 3 hearts of each genotype), two-tailed Student's t-test. Scale bar: 50μm.

Suppl. Figure 4. B1KO hearts display trabecular and compact zone specification defects.

(A-B) *Hey2* level was decreased, but *Bmp10* mRNA level was increased in the B1KO hearts (n=6, every 2 hearts with the same genotype were combined for Q-PCR), two-tailed Student's t-test. (C&D) Smad1/5/8 showed more phosphorylation in cardiomyocytes of the B1KO hearts, indicated by p-Smad1/5/8, a readout for *Bmp10*. (E&F) *Nppa* is enriched in the trabecular zone in control hearts but is also highly expressed in the compact zone of B1KO hearts. (G) The percentage of P21 expressing cardiomyocytes is significantly increased in B1KO hearts (n=6, 6 sections from 3 hearts of each genotype), two-tailed Student's t-test. (H&I) The trabecular cardiomyocytes, sarcomeric array formation, are more organized than the compact cardiomyocytes in control hearts. In the B1KO hearts, the compact cardiomyocytes formed a sarcomeric array, indicating an early differentiation defect. (J&K) Sarcomeric array formation in the compact zone of B1KO hearts is more organized than in the control hearts but less organized than in the cells in the trabecular zone of the control based on EM. Scale bar: 50μm in C-F, 20μm in H&I.

Suppl. Figure 5. Unedited western blot data for Figure 3.

Gel 1 (left to right): Control (wild type, n=3), B1KO (n=3). Gel 2 (left to right): Control (wild type, n=3), B1KO (n=3).

Suppl. Figure 6. Cardiomyocyte proliferation rate is reduced at E9.5&E10.5, but not E8.5 in B1KO hearts compared to control littermates.

(A-D) Based on BrdU pulse labeling, the cardiomyocytes in E10.5 B1KO hearts displayed a lower

proliferation rate than the control (n=5), two-tailed Student's t-test. (E-G) The cardiomyocyte proliferation rate in E8.5 B1KO hearts did not show an obvious difference from the control (n=5), two-tailed Student's t-test. (H&I) The asymmetric cell division studies were further confirmed using *Nkx2.5*$^{Cre/+}$*; Itgb1*$^{fl/+}$*; mTmG* (control) and *Nkx2.5*$^{Cre/+}$*; Itgb1*$^{fl/fl}$*; mTmG* (B1KO) hearts, in which cell boundary was marked by the membrane-localized GFP. Scale bar: 50μm in A&B, E&F, 10μm in H&I.

Suppl. Figure 7. Unedited western blot data for Figure 6.
Gel 1 (left to right): Control (wild type, n=3), B1KO (n=3). Gel 2 (left to right): Control (wild type, n=3), B1KO (n=3).

Suppl. Figure 8. Schematic pictures illustrate the β1 integrins regulating cellular behaviors and organization during ventricular wall formation.
(A&B) β1 was asymmetrically distributed in the cardiomyocytes, enriched in the luminal side of the cardiomyocytes in the E9.5 control heart. (C) β1, Fn, and laminin 411 are asymmetrically distributed to the luminal side of the myocardium and establish a polarized network frame, providing a scaffold for the cardiomyocytes to be stabilized in the myocardium. Loss of β1 in the myocardium caused the failure of forming the polarized network frame.
